# Big data analytics and scRNA-seq in human aortic aneurysms and dissections: role of endothelial MerTK

**DOI:** 10.7150/thno.103851

**Published:** 2025-01-01

**Authors:** Shijie Liu, Jinzi Wu, Oishani Banerjee, Bingzhong Xue, Hang Shi, Zufeng Ding

**Affiliations:** Department of Biology, Georgia State University, Atlanta, GA, 30303, USA.

**Keywords:** aortic aneurysms and dissections, endothelial cells, MerTK, big data analytics, scRNA-seq

## Abstract

**Rationale:** Aortic aneurysms and dissections (AAD) cause more than 10,000 deaths in the United States each year. However, there are no medications that can effectively prevent the pathogenesis of AAD. MER proto-oncogene tyrosine kinase (MerTK) is a key receptor for efferocytosis, a process for the clearance of apoptotic cells. Here, we mainly focused on ascending aortic aneurysms and dissections (AAAD) and investigated the role of endothelial MerTK in AAAD progression.

**Methods:** Single-cell RNA sequencing (scRNA-seq) analysis in human AAAD samples and RNA-seq big data analytics, combined with our unique *MerTK^flox/flox^/Tie2^Cre^* mouse model with MerTK deficiency in endothelial cells (ECs), were applied to define the role of endothelial MerTK in AAAD.

**Results:** Through comparative analyses of scRNA-seq in human AAAD (communications of ECs with other cells) and comprehensive big data analytics including about 600,000 cross analyses, we found that the expression of endothelial MerTK is significantly inhibited in human AAAD, resulting in decreased ability of ECs to engulf antigen presenting cells, phagocytes, leukocytes, blood cells and myeloid cells. Our *in vivo* data showed a significantly higher incidence of AAAD in MerTK*^flox/flox^/Tie2^Cre^* mice compared to that of their littermate controls of MerTK*^flox/flox^* mice (100% vs. 11.1%). MerTK deficiency in ECs induces both endothelial dysfunction and SMC phenotypic alterations, subsequently promoting AAAD development.

**Conclusions:** Our findings indicate that endothelial MerTK impairment and subsequent endothelial dysfunction and SMC phenotypic alterations represent novel mechanisms promoting AAAD.

## Introduction

Ascending aortic aneurysms and dissections (AAAD) are a potentially lethal condition associated with endothelial dysfunction, smooth muscle cell (SMC) phenotypic alterations, and inflammatory and immune responses 1. The vascular endothelium is a single layer of endothelial cells (ECs) that play an important physiological role in vascular homeostasis, including maintenance of blood fluidity, production of vasoactive molecules, and regulation of proinflammatory immune responses 2. Endothelial dysfunction is characterized by decreased expression of endothelial NO synthase (eNOS) and production of endothelial nitric oxide (NO) as well as increased oxidative stress and endothelial inflammation.^3^ Endothelial dysfunction is also related to increased accumulation of apoptotic cells and mitochondrial dysfunction 3,4. Aortic SMCs possess remarkable phenotypic plasticity that enable them to rapidly adapt to environmental changes 5. In a healthy vessel wall, SMCs maintain a contractile phenotype and remain quiescent. However, under pathological conditions, they lose this contractile phenotype and gain an inflammatory and synthetic phenotype. Synthetic SMCs are characterized by a decreased expression of contractile proteins (e.g. SM22α - smooth muscle 22α and α-SMA - α-Smooth muscle actin), along with an increased secretion of growth factors, pro-inflammatory cytokines, and extracellular matrix (ECM) proteins 6. Endothelial dysfunction and SMC phenotypic alterations are common features in AAAD 7,8.

AAAD can be directly triggered by several mediators, such as proinflammatory cytokines, oxidative stress, and accumulation of apoptotic cells, all of which are involved in efferocytosis 9. Efferocytosis is a precisely orchestrated process in which phagocytes are recruited to the apoptotic tissue, where they recognize, engulf, and clear dying cells in an immunologically silent manner 10. Efferocytosis is essential for maintaining tissue homeostasis and its impairments can result in the buildup of apoptotic cells, which in turn contributes to the development of cardiovascular diseases 10. Mer Tyrosine Kinase (MerTK) is the main receptor of efferocytosis in phagocytes 11. Increasing evidence has shown that ECs are capable of engulfing apoptotic cells 12-16. Our recent studies showed that primary aortic ECs have a high ability to perform efferocytosis via MerTK and play an important role in vascular aging and atherosclerosis, the diseases that are closely associated with AAAD 14-16. Inhibition of endothelial MerTK impairs efferocytosis, leading to the accumulation of apoptotic cells, secondary post-apoptotic necrosis, and pro-inflammatory responses 14-16. More importantly, ECs interact with the neighboring SMCs, potentially influencing the SMC phenotypic alteration 17. However, the mechanisms underlying AAAD are largely unknown and the role of endothelial MerTK in the pathogenesis of AAAD has yet to be identified.

Big data analytics provides an effective approach to form scientific hypothesis and uncover the shared signaling pathways and underlying mechanisms across different projects with similar pathological conditions. The single-cell RNA sequencing (scRNA-seq) offers another method to explore the transcriptomic changes at the single-cell level. Here, we mainly focused on cell-cell communication between ECs and other cells, investigating the role of endothelial MerTK in the development of AAAD. Both the scRNA-seq analysis in human AAAD and our big data analytics indicate that impaired endothelial MerTK and subsequent endothelial efferocytosis may play a key role in AAAD development. We further validated the findings derived from the scRNA-seq analysis and big data analytics using our unique mouse model with EC-specific MerTK knockout (*MerTK^flox/flox^/Tie2^Cre^*). Our data provides direct evidence that endothelial MerTK deficiency promotes endothelial dysfunction and SMC phenotypic alterations, accelerating AAAD development.

## Methods

**Big data analytics and scRNA-seq in human AAAD.** The big data analytics and scRNA-seq data in human AAAD were derived from QIAGEN Ingenuity Pathway Analysis (IPA) and QIAGEN OmicSoft Land Explorer (OLE), which incorporate data from GEO (Gene Expression Omnibus), SRA (Sequence Read Archive), and ArrayExpress. For the aortic aneurysm screening in scRNA-seq data from human samples, we set 'aortic aneurysm' as the key words in IPA Datasets and Analyses. Among 1522 projects of aortic aneurysm, we filtered the data with the key words of 'ascending aorta' in Case.Tissue. We identified the scRNA-seq data in human samples from IPA Datasets (GSE# 155468), including 8 patients with aortic aneurysm (4 males and 4 females with age of 56-75) and controls without aortic aneurysm (1 male and 2 females with age of 61-63) 8. The detailed patient's information (e.g., medical conditions and therapies) and scRNA-seq preparation was described by Li *et al.* 8. IPA# [100-107 aortic aneurysm (ascending aorta) NA 22082-22089] were extracted for the comparison analysis between EC with other cell types, including **1**) EC vs macrophages with IPA# 100-aortic aneurysm (ascending aorta) NA 22082. The downregulated and upregulated log2 cutoff were set as -0.7193 and 0.5677 respectively. **2**) EC vs mast cell with IPA# 101-aortic aneurysm (ascending aorta) NA 22083. The downregulated and upregulated log2 cutoff were set as -1.0202 and 0.52 respectively. **3**) EC vs mesenchymal stem cell (MSC) with IPA# 102-aortic aneurysm (ascending aorta) NA 22084. The downregulated and upregulated log2 cutoff were set as -0.6047 and 0.4574 respectively. **4**) EC vs monocyte with IPA# 103-aortic aneurysm (ascending aorta) NA 22085. The downregulated and upregulated log2 cutoff were set as -1.6799 and 0.7982 respectively. **5**) EC vs NK cell with IPA# 104-aortic aneurysm (ascending aorta) NA 22086. The downregulated and upregulated log2 cutoff were set as -1.2438 and 0.4992 respectively. **6**) EC vs plasma B cell with IPA# 105-aortic aneurysm (ascending aorta) NA 22087. The downregulated and upregulated log2 cutoff were set as -0.1635 and 0.9883 respectively. **7**) EC vs SMC with IPA# 106-aortic aneurysm (ascending aorta) NA 22088. The downregulated and upregulated log2 cutoff were set as -0.8633 and 0.4413 respectively. **8**) EC vs unassigned cell with IPA# 107-aortic aneurysm (ascending aorta) NA 22089. The downregulated and upregulated log2 cutoff were set as -1.4025 and 0.5932 respectively. For these analyses, the patients were limited to male, non-smokers and non-Hispanic; the cells were isolated from ascending aorta; and OmicsoftGenCode.V33 was used as Gene Model ID. For big data analytics, we set 'aortic aneurysm' as the key words in IPA Pathways and Lists. We identified the signaling pathways of aortic aneurysm, aortic dissection, abdominal aortic aneurysm and thoracic aortic aneurysm. The big data analytics include 160790 cross analyses for aortic aneurysm, 155028 cross analyses for aortic dissection, 160097 cross analyses for abdominal aortic aneurysm, and 127340 cross analyses for thoracic aortic aneurysms.

Based on QIAGEN IPA citation guidelines, a data set containing gene identifiers and corresponding data measurement values was uploaded into the application. Each identifier was mapped to its corresponding entity in QIAGEN's Knowledge Base. Network Eligible molecules were overlaid onto a global molecular network developed from information contained in the QIAGEN Knowledge Base. Networks of Network Eligible Molecules were then algorithmically generated based on their connectivity. The Diseases & Functions Analysis identified the biological functions and/or diseases that were most significant from the data set. Molecules from the dataset that were associated with biological functions and/or diseases in the QIAGEN Knowledge Base were considered for the analysis. A right-tailed Fisher's Exact Test was used to calculate a p-value determining the probability that each biological function and/or disease assigned to that data set is due to chance alone. A z-score was calculated to indicate the likelihood of an increase or decrease of that disease or function. There are about 1500 diseases, phenotypes, and function pathways created by machine learning (ML) in the QIAGEN Knowledge Base. These ML Disease Pathways show key molecules that impact a single disease and its associated phenotypes, which may represent novel participants in the disease or its etiology.

**Animals.** The *MerTK^flox/flox^* mice and Tie2-Cre mice on the C57BL/6J background, our unique designed project of CKOCMP-17289-Mertk-B6J-VA were generated by Cyagen US (Santa Clara, CA) and housed in the Division of Laboratory Animal Medicine at our institution. All experimental procedures were performed in accordance with protocols approved by the Institutional Animal Care and Use Committee and conformed to the Guidelines for the Care and Use of Laboratory Animals published by the US National Institutes of Health. The mice were maintained under a 12-hour light/12-hour dark cycle at a controlled temperature of 21 ± 1 ºC, with ad libitum access to water and a standard laboratory diet. *MerTK^flox/flox^/Tie2^Cre^* mice with MerTK conditional knockout in ECs were generated by crossing* MerTK^flox/flox^* mice with Tie2-Cre mice. The 8-week-old male *MerTK^flox/flox^/Tie2^Cre^* mice and their littermate controls (*MerTK^flox/flox^*) were infused with saline or Ang II (1000 ng·kg^-1^·min^-1^) for 7 days to establish the AAAD model. After mice were euthanized by CO_2_ asphyxiation, the ascending aorta tissues were carefully dissected from surrounding tissue, fixed with 10% neutral buffered formalin solution (Sigma, HT501128), and embedded in paraffin for further immunohistochemical analysis.

**Genotyping.** Genomic DNA was extracted from the tails of the mice using Platinum™ Direct PCR Universal Master Mix (Invitrogen) according to the manufacturer's instructions. PCR was performed with a 20 μL reaction volume containing 10 µL 2x Platinum™ Direct PCR Universal Master Mix, 1-2 µL sample supernatant, 18-19 µL nuclease-free water, and 0.2 µM primers for *MerTK^flox/flox^* gene of F1: 5'-TTACCTCATGGTATCTGCTGGCTA-3'; R1: 5'-ACCACTTTCTCTTTGGTTGGAGT-3' and *Tie2-Cre* gene of F1: 5'-CCCTGTGCTCAGACAGAAATGAGA-3'; R1: 5'-CGCATAACCAGTGAAACAGCATTGC-3'. The products were separated by 1.5 % agarose gel electrophoresis and visualized with SYBR™ Safe DNA Gel Stain (Invitrogen) by Syngene™ NuGenius Gel Immaging System.

**Immunofluorescent staining.** Immunofluorescent staining was performed according to the protocol of 'Immunofluorescent Staining of Paraffin-Embedded Tissue (Novus Biologicals)'. The information of antibodies is shown as follows: TLR4 was from Santa Cruz (sc-293072); α-SMA (Cat. 19245), SM22-α (Cat. 40471), CloA1 (Cat. 39952), SOX9 (Cat. 82630) and NF-κB (Cat. 8242) were from Cell Signaling.

**Statistical analysis.** An unpaired Student's t-test was used to determine statistical significance between two groups. Dunnett's one-way ANOVA was used for multiple comparisons between disease types and normal control. Data was analyzed with GraphPad Prism 9.4.1 and summarized as the mean ± SD. P < 0.05 was considered statistically significant.

## Results

**Comparative analysis of EC and adjacent cell communications in human AAAD based on scRNA-seq.** ECs, SMCs and immune cells (e.g., macrophages and NK cells) play important roles in the pathogenesis of AAAD 1. Aortic aneurysm is a chronic inflammatory disease featured by infiltration and activation of inflammatory cells such as macrophage within adventitial and medial layers of the aortic walls 18. Macrophages have been shown to accumulate in aneurysmal lesions of aortic walls from both human and animal models of aortic aneurysm 19. NK cells, a key player of the innate immune system, are cytotoxic lymphocytes that contribute to aortic aneurysm by inducing matrix metalloproteinases (MMPs) in SMCs and macrophages, leading to aortic wall damage 20-22. Importantly, communication between ECs and SMCs, macrophages or NK cells may play a central role in AAAD development. MerTK is the main receptor for efferocytosis in aortic ECs. Endothelial MerTK deficiency promotes the accumulation of apoptotic cells, increased oxidative stress and pro-inflammatory responses 14-16, all of which are the key inducers for SMC phenotypic alterations 23 and the activation of macrophages and NK cells 24. To reveal the specific role of ECs in the pathogenesis of AAAD, we performed comparative analyses between ECs and other cells using scRNA-seq data from human AAAD patients. The scRNA-seq data were downloaded from the QIAGEN IPA database that offers access to >600,000 omics samples and >100,000 expression datasets via QIAGEN OLE for IPA and analysis match.

**Figure 1:**
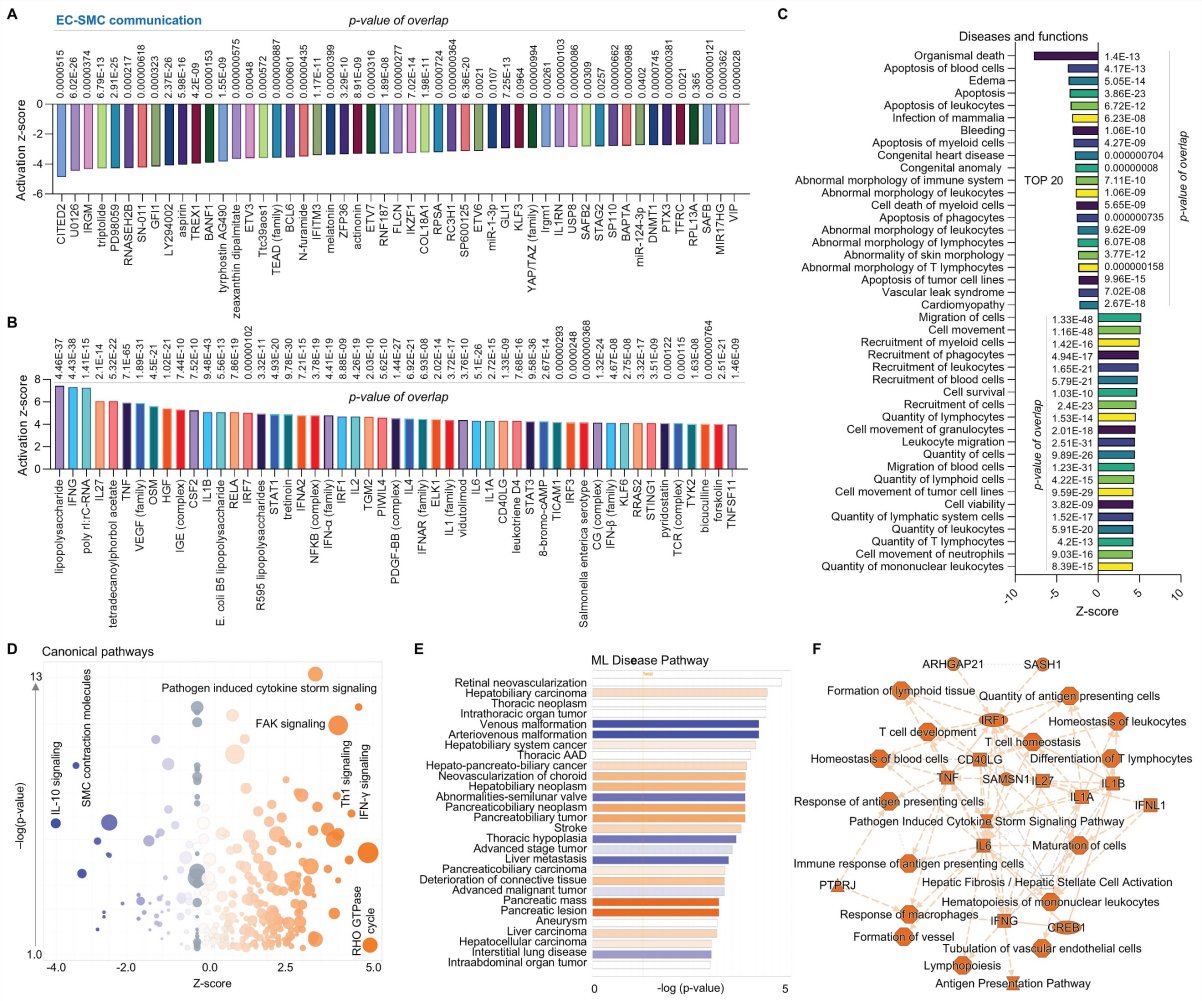
** Comparative analysis of EC-SMC communication at single cell level in ascending aorta of human AAAD.** (**A**-**B**) The top 50 downregulated and upregulated upstream regulators based on activation of z-score. (**C**) Diseases and functions analysis based on activation of z-score. (**D**) The volcano canonical pathways based on activation of z-score. Blue: negative value. Orange: positive value. Grey: no activity pattern. Size is based on the number of genes that overlap the pathway. (**E**) Machine learning (ML) disease pathway predicts activated signaling pathways (orange) and inhibited signaling pathways (bule). (**F**) Graphical summary of scRNA-seq data shows upregulated signaling (orange) and downregulated signaling (blue). QIAGEN Ingenuity Pathway Analysis [IPA: 106-aortic aneurysm (ascending aorta) NA 22088] and QIAGEN OmicSoft Land Explorer (OLE) were used to analyze scRNA-seq in ascending aorta from human AAD patients.

First, we performed comparative analysis of ECs and SMCs using scRNA-seq in human AAAD samples (IPA: 106-aortic aneurysm-ascending aorta NA 22088 and GSE# 155468). The Upstream Regulator Analysis is based on expected causal effects between upstream regulators and targets. IPA Upstream Regulator Analysis is designed to identify upstream regulators and predict whether they are activated or inhibited, given the observed gene expression changes in the experimental dataset. Therefore, we first analyzed Upstream Regulator Analysis, assessing the direct communication between ECs and SMCs. We identified the top 50 of both inhibited genes (e.g., CITED2- Cbp/p300-interacting transactivator 2, IRGM-immunity-related GTPase family M protein, and GFI1- a zinc finger protein, **Figure 1A**) and activated genes (e.g., VEGF-vascular endothelial growth factor family, NF-κB family, and IL1 family, **Figure 1B**). The disease and functional analysis show that, compared to SMCs, ECs in AAAD are positively associated with activation of cell migration, recruitment of immune cells (e.g., myeloid cells, phagocytes, leukocytes and blood cells), and increased quantity of lymphoid cells, lymphatic system cells, leukocytes and T lymphocytes (**Figure 1C**). Conversely, ECs are negatively associated with organismal death, apoptosis of cells (e.g., blood cells, leukocytes, myeloid cells), and abnormal morphology of a number of immune cells. **Figure 1D** presents canonical pathways, highlighting a well-characterized metabolic and cell signaling pathway. This includes inhibited pathways such as IL-10 signaling and SMC contraction molecules, and activated pathways such as pathogen induced cytokine storm signaling, FAK-focal adhesion kinase signaling, Th1 signaling, and IFN-γ signaling and Rho GTPase cycle. Machine learning (ML) disease pathway analysis revealed both activated diseases (e.g., hepatobiliary carcinoma, hepato-pancreato-biliary cancer and pancreatic lesion) and inhibited diseases (e.g., venous malformation, arteriovenous malformation and abnormalities-semilunar valve) (**Figure 1E**). The graphical summary shows that, compared to SMCs, ECs exhibit an activated inflammatory response marked by increased production of a panel of pro-inflammatory cytokines or chemokines, including TNF-tumor necrosis factor, interleukin (IL)-6, IL-1A, IL-1B and interferon (IFN)-γ (**Figure 1F**), which appears to induce an immune activity that impact T cell development, response of macrophages, differentiation of T lymphocyte and response of antigen presenting cells. In summary, compared to SMCs, ECs appear to activate various inflammatory and immune responses while inhibit cell apoptosis and abnormal cell morphology, indicating the protective role of ECs as the first barrier at the blood-tissue interface in AAAD.

**Figure 2:**
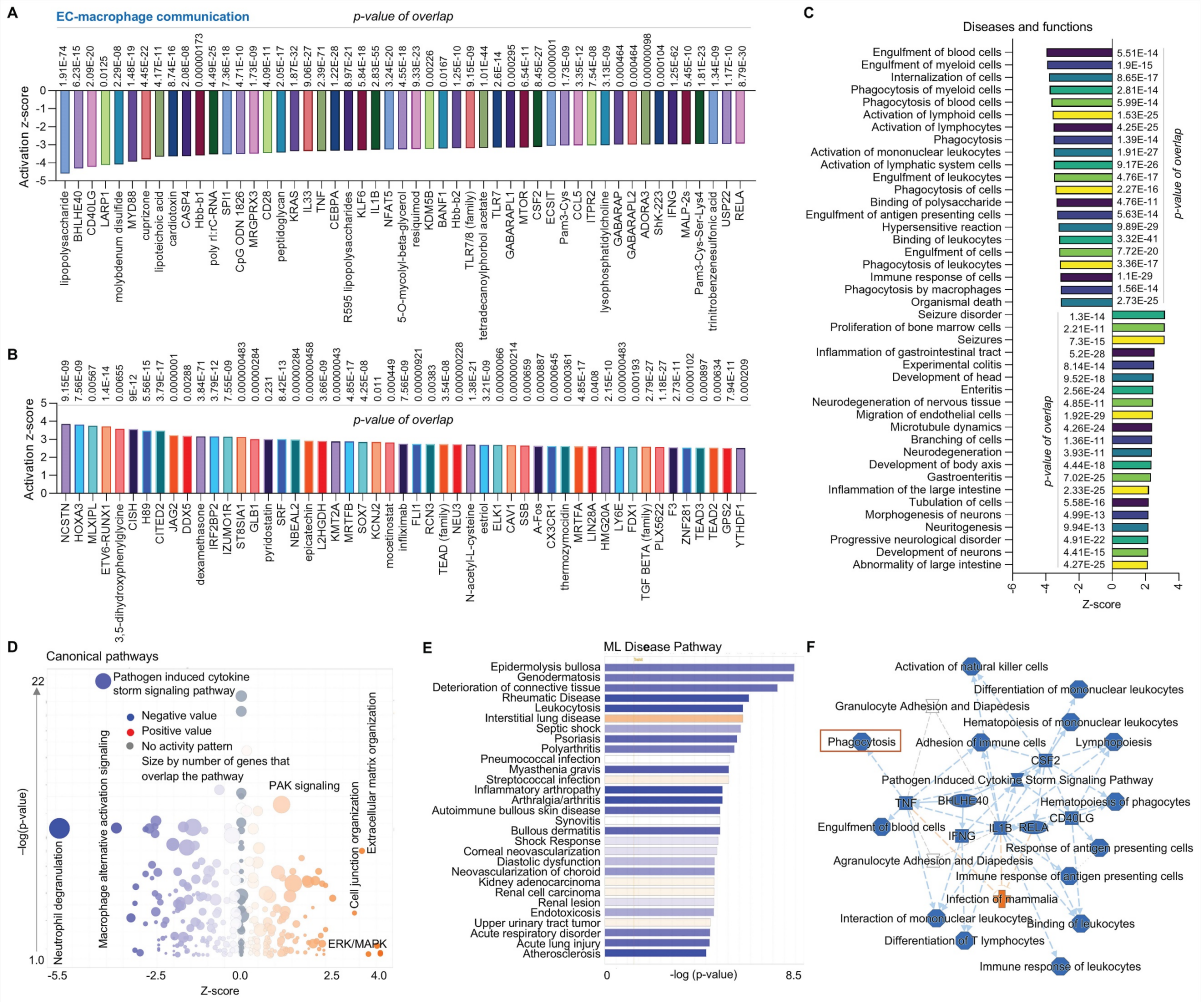
** Comparative analysis of EC-macrophage communication at single cell level in ascending aorta of human AAAD.** (**A**-**B**) The top 50 downregulated or upregulated upstream regulators based on activation of z-score. (**C**) Diseases and functions analysis based on activation of z-score. (**D**) The volcano canonical pathways based on activation of z-score. Blue: negative value. Orange: positive value. Grey: no activity pattern. Size is based on the number of genes that overlap the pathway. (**E**) Machine learning (ML) disease pathways (orange: upregulated; blue: downregulated). (**F**) Graphical summary of scRNA-seq data (orange: upregulated; blue: downregulated). QIAGEN Ingenuity Pathway Analysis [IPA: 100-aortic aneurysm (ascending aorta) NA 22082] and QIAGEN OmicSoft Land Explorer (OLE) were used to analyze scRNA-seq in ascending aorta from human AAD patients.

Second, we conducted a comparative analysis of ECs and macrophages using scRNA-seq in human AAAD tissues. **Figure 2A** and **2B** focus on upstream pathways of both inhibited signaling (e.g., CD40LG-a type II transmembrane glycoprotein, MYD88-downstream of TLR4 signaling, and a series of pro-inflammatory factors) and activated signaling (e.g., NSCTN-nicastrin, HOXA3-a homeobox protein and TGF-β family-an inflammation factor).** Figure 2C** outlines an analysis of diseases and functions, revealing activated bone marrow cells proliferation and EC migration, as along with inhibited engulfment of cells (e.g., blood cells, myeloid cells and leukocytes) and immune response of cells. **Figure 2D** presents canonical pathways including both downregulated signaling (e.g., neutrophil degranulation, macrophage alternative activation signaling, and pathogen induced cytokine storm signaling) and upregulated signaling (e.g., PAK-p21 activated kinases, ERK/MAPK and cell junction organization). Using ML analysis, **Figure 2E** depicts disease pathways including increased interstitial lung disease and decreased diseases (e.g., deterioration of connective tissue, inflammatory arthropathy, and endotoxicosis). **Figure 2F** provides a graphical summary that shows both activated signaling (e.g., infection of mammalian) and a series of inhibited signaling (e.g., pro-inflammatory factors, phagocytosis and cell differentiation of mononuclear leukocytes). Notably, scRNA-seq analysis for comparison between ECs and macrophages supports our recent findings on EC efferocytosis,^14-16^ providing the rationale for using the animal model (*MerTK^flox/flox^/Tie2^Cre^* mice, MerTK conditional knockout in ECs) for *in vivo* validation of human scRNA-seq data. In summary, compared to macrophages, ECs secrete lower levels of pro-inflammatory cytokines or chemokines and have a lower ability to perform engulfment and phagocytosis.

**Figure 3:**
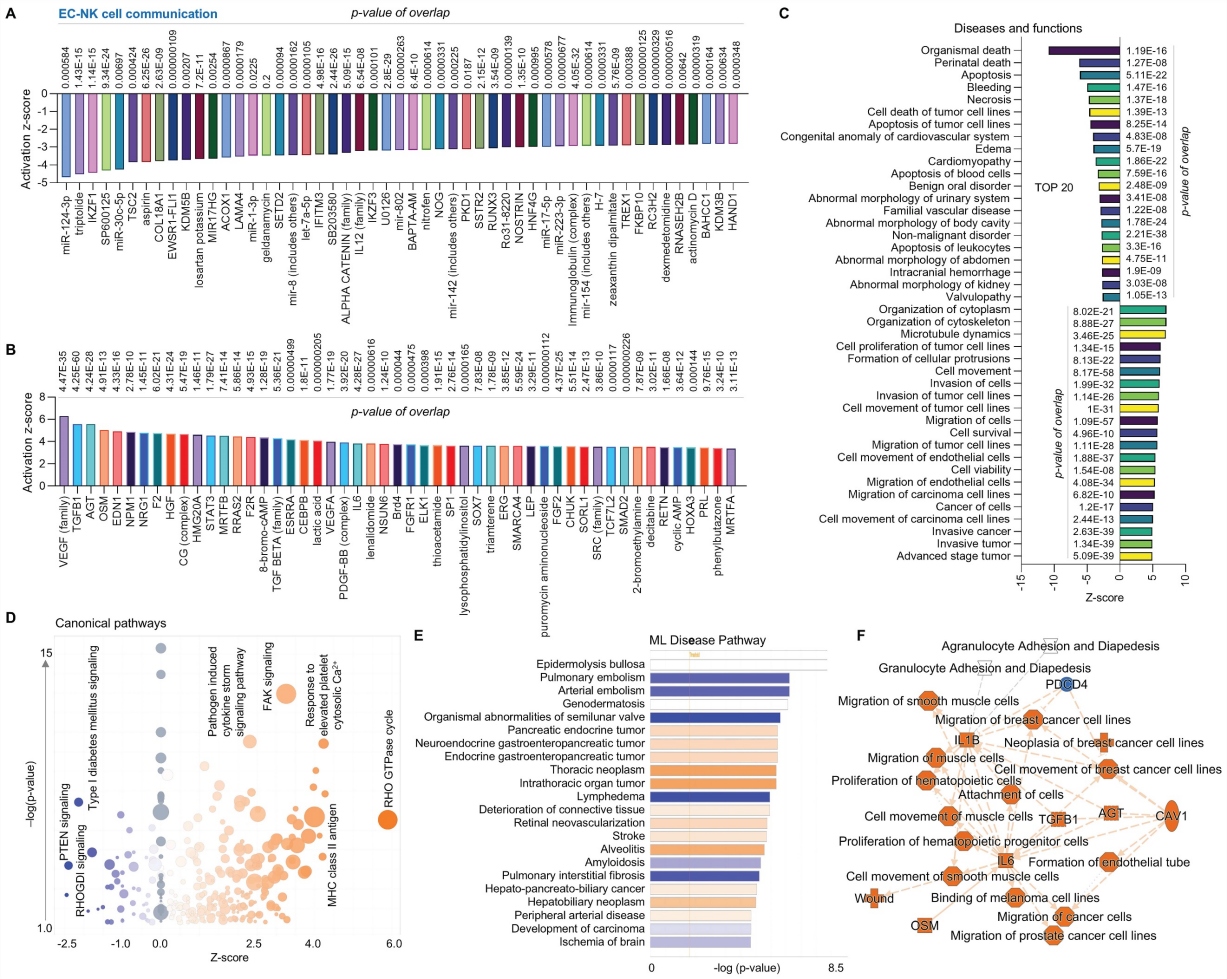
** Comparative analysis of EC-NK cell communication at single cell level in ascending aorta of human AAAD.** (**A**-**B**) The top 50 downregulated or upregulated upstream regulators based on activation of z-score. (**C**) Diseases and functions analysis based on activation of z-score. (**D**) The volcano canonical pathways based on activation of z-score. Blue: negative value. Orange: positive value. Grey: no activity pattern. Size is based on the number of genes that overlap the pathway. (**E**) Machine learning (ML) disease pathways (orange: upregulated; blue: downregulated). (**F**) Graphical summary of scRNA-seq data (orange: upregulated; blue: downregulated). QIAGEN Ingenuity Pathway Analysis [IPA: 104-aortic aneurysm (ascending aorta) NA 22086] and QIAGEN OmicSoft Land Explorer (OLE) were used to analyze scRNA-seq in ascending aorta from human AAD patients.

Third, we performed a comparison analysis between ECs and NK cells using scRNA-seq in human AAAD samples. In upstream pathway analysis, ECs exhibit inhibited expression of microRNAs (e.g. miR-124-3p, miR-30c-5p, miR1-3p, and let-7a-5p) and other genes as shown in the figure and increased expression of VEGF family, TGF-β1 and VEGF-A (**Figure 3A**-**3B**). The analysis of diseases and functions indicates that ECs have upregulated organization of cytoplasm and cytoskeleton and cell movement (e.g., ECs and tumor cells) along with downregulated cell apoptosis, cell necrosis and cell death (**Figure 3C**). The canonical pathway analysis shows inhibition of signaling of PTEN-phosphatase and tensin homolog, RHOGDI-Rho guanine nucleotide dissociation inhibitors and type I diabetes mellitus in ECs, with activation of signaling of RHO GTPase cycle, FAK and pathogen induced cytokine storm pathway (**Figure 3D**). The ML disease pathway analysis shows that ECs are associated with inhibition of arterial embolism, organismal abnormalities of semilunar valve, and pulmonary interstitial fibrosis and activation of thoracic neoplasm, intrathoracic organ tumor, and hepatobiliary neoplasm (**Figure 3E**). We also found that, compared to NK cells, ECs display elevated production of various pro-inflammatory cytokines or chemokines (e.g., IL-1B, TGF-B1, and IL-6), increased cell movement (e.g., SMCs, muscle cells and cancer cell lines), and induced formation of endothelial tube, along with inhibition of PDCD4 (programmed cell death 4) as shown in **Figure 3F**. In summary, compared to NK cells, ECs play a predominant role in immune responses and protect other cells from damage.

**Figure 4:**
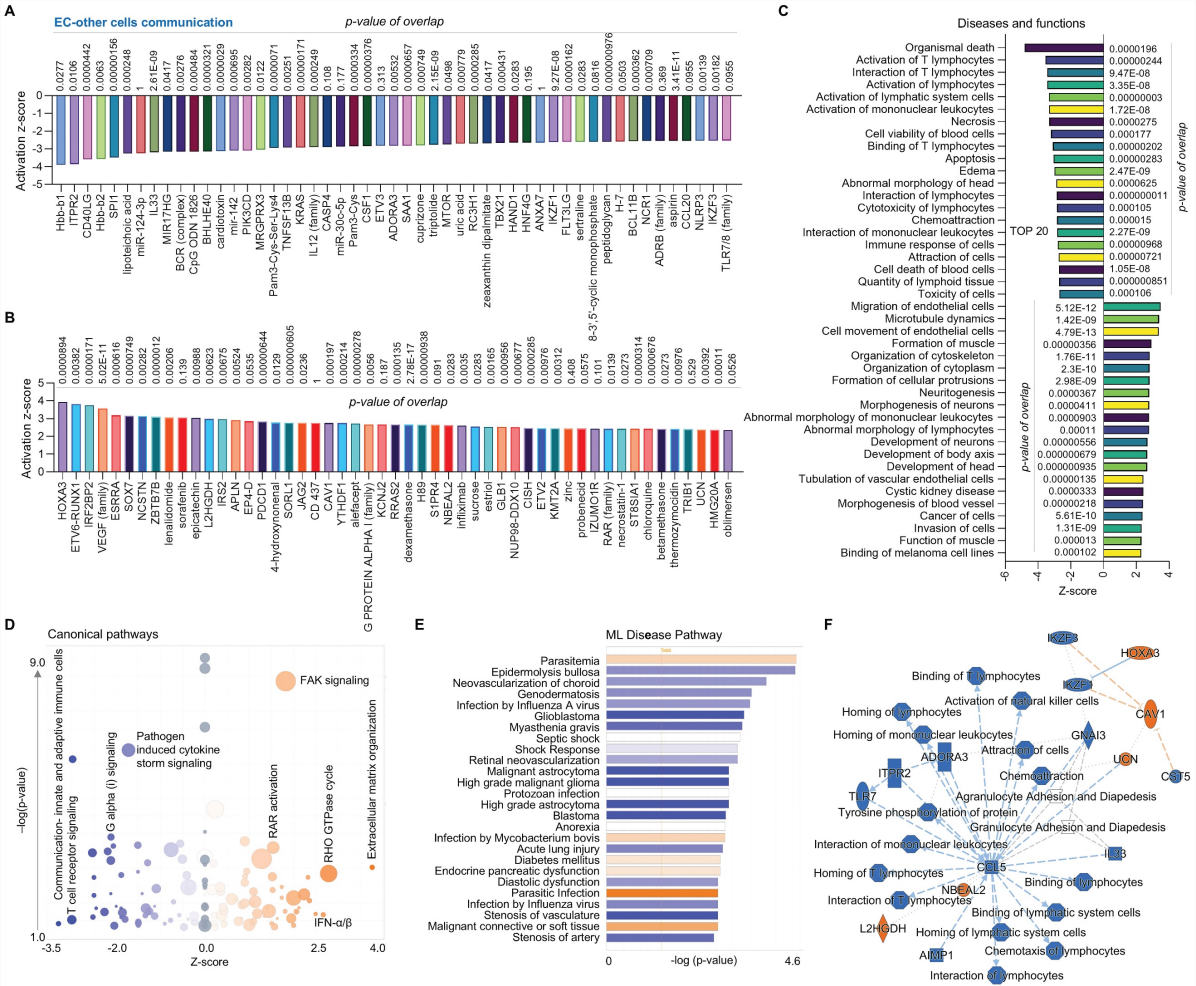
** Comparative analysis of EC clusters with others in ascending aorta of human AAAD.** (**A**-**B**) The top 50 downregulated or upregulated upstream regulators based on activation of z-score. (**C**) Diseases and functions analysis based on activation of z-score. (**D**) The volcano canonical pathways based on activation of z-score. Blue: negative value. Orange: positive value. Grey: no activity pattern. Size is based on the number of genes that overlap the pathway. (**E**) Machine learning (ML) disease pathways (orange: upregulated; blue: downregulated). (**F**) Graphical summary of scRNA-seq data (orange: upregulated; blue: downregulated). QIAGEN Ingenuity Pathway Analysis [IPA: 166-aortic aneurysm (ascending aorta) NA 22148] and QIAGEN OmicSoft Land Explorer (OLE) were used to analyze scRNA-seq in ascending aorta from human AAD patients.

Finally, we compared the EC cluster with other cells using scRNA-seq in human AAAD samples. The upstream pathway analysis shows inhibited signaling (e.g., hbb-b1&2-hemoglobin, ITPR2-inositol 1,4,5-trisphosphate receptor type 2, and several miRs, **Figure 4A**) and activated signaling (e.g., HOXA3, VEGF family and SOX 7- SRY box transcription factor 7, **Figure 4B**). Additionally, the analysis of diseases and functions shows upregulated pathways, including EC migration, EC tubulation, and cell morphology abnormalities, along with inhibited pathways, including T lymphocytes activation, cell apoptosis and immune response (**Figure 4C**). The canonical pathway analysis shows that ECs have downregulated communications between innate and adaptive immune cells, T cell receptor signaling, and pathogen induced cytokine storm signaling while having upregulated extracellular matrix organization, FAK signaling and RHO GTPase cycle (**Figure 4D**). The ML disease pathway analysis indicates both inhibited pathways, such as stenosis of artery, and activated pathways, such as diabetes mellitus (**Figure 4E**). For the summary signaling as shown in **Figure 4F**, ECs have increased expression of L2HGDH (L-2-hydroxyglutarate dehydrogenase, a mitochondrial protein), NBEAL2 (neurobeachin like 2), UCN (urocortin), CAV1 (caveolin 1) and HOXA3. By contrast, ECs have various down-regulated signaling pathways, including CCL5 (C-C motif chemokine ligand 5, a chemokine related immune response), as well as homing, binding and interaction of cells (e.g., T lymphocytes and mononuclear leukocytes), and activation of NK cells. Interestingly, CCL5 appears to play a central role in EC-mediated pathways. In summary, compared to other cells, ECs play an important role in maintaining vascular stability and adapting various inflammatory and immune responses.

**Figure 5:**
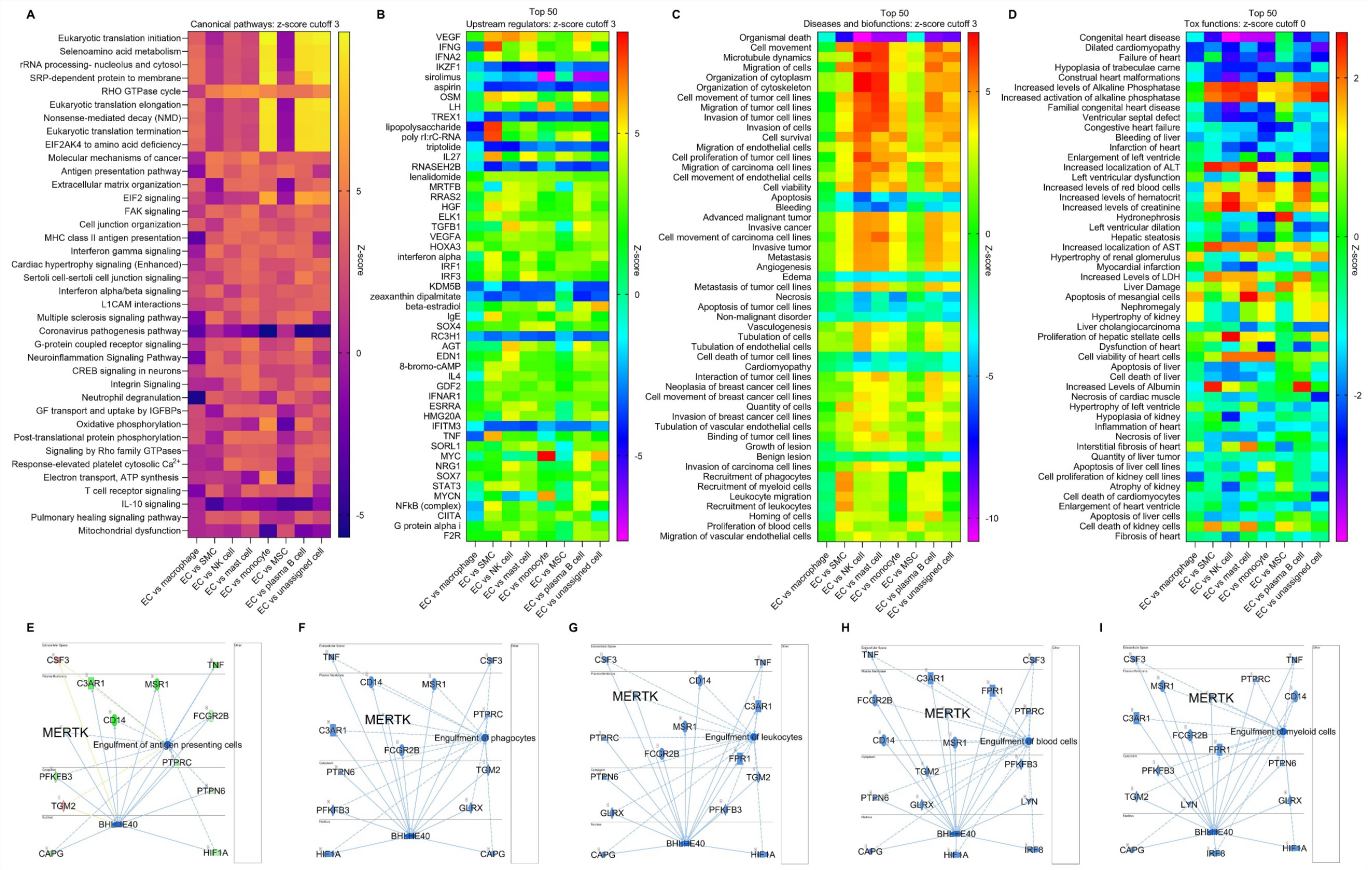
**Multi-comparative analysis of ECs with other cells in ascending aorta of human AAAD.** (**A**) Heatmap for the canonical pathways with comparative analysis between ECs and other cells, including macrophages, SMCs, NK cells, mast cells, monocytes, MSCs, plasma B cells and other unassigned cells. (**B**) Comparative analysis for top 50 upstream regulators based of activation of z-score. (**C**) Comparative analysis for diseases and biofunctions within top 50 based on activation of z-score. (**D**) Comparative analysis for top 50 tox functions based on activation of z-score. (**E-I**) Signaling pathways for EC-mediated cell engulfment are involved in MerTK. Red: increased measurement. Green: decreased measurement. Orange: predicted activation. Blue: predicted inhibition. In multi-comparative analysis, MerTK was set as the target molecule in dataset and sorted by consistency score. QIAGEN Ingenuity Pathway Analysis [IPA: 100-107 aortic aneurysm (ascending aorta) NA 22082-22089] and QIAGEN OmicSoft Land Explorer (OLE) were used to analyze scRNA-seq in ascending aorta from human AAD patients.

**Multi-comparison of ECs and other cells using scRNA-seq in human AAAD.** To further investigate the role of ECs in AAAD development, we performed multi-comparative analysis involving ECs, macrophages, SMCs, NK cells, mast cells, monocytes, mesenchymal stem cells (MSCs), plasma B cells and all other unassigned cells. Mast cells, a resident cell in connective tissues, play an important role in inflammatory responses and immunity 22. Monocytes, circulating leukocytes derived from the bone marrow, are a critical component of the innate immune response to inflammation 25. MSCs are multipotent stromal cells that help vascular self-repair after injury 26. Plasma B cells secrete specific immunoglobulins or antibodies 27. These immune cells including monocytes, macrophages, SMCs, NK cells, mast cells, MSCs and plasma B cells have been identified as key players in the pathogenesis of aortic aneurysm 1. First, we summarized the canonical pathways in **Figure 5A**, which shows the significantly changed signaling pathways between ECs and other cells, such as RHO GTPase cycle, EIF2 (eukaryotic initiation factor 2) signaling, IL-10 signaling and mitochondrial dysfunction. Second, an analysis of the top 50 upstream regulators indicated that various genes (e.g., VEGF, TNF-γ, IL27, MYC-myelocytomatosis oncogene, and NF-κB) may play important roles in the progression of AAAD (**Figure 5B**). Third, our disease and biofunctional analysis show that ECs play a predominant role in the pathways responsible for cell survival and death (e.g., apoptosis and necrosis), cell migration (e.g., ECs and cancer cells) and cell recruitment (e.g., phagocytes, myeloid cells and leukocytes) in AAAD (**Figure 5C**). Finally, we explored the EC-mediated toxic functions, predicting that ECs may play a key important role in cardiac dysfunction (e.g., congenital heart disease and myocardial infarction), increased levels of LDH (lactate dehydrogenase, a sign of tissue damage or disease), and dysfunctions of liver and kidney (**Figure 5D**). More importantly, consistent with our previous findings on efferocytosis in aortic ECs 14-16, we provided new evidence that EC-mediated engulfment (e.g., antigen presenting cells, phagocytes, leukocytes, blood cells and myeloid cells) is significantly inhibited in AAAD, possibly due to decreased levels of MerTK in ECs (**Figure 5E**-**5I**). In summary, our multi-comparative analysis further validates the key role of ECs in AAAD. The novel findings on decreased capacity of EC engulfment related to MerTK provides the direct rationale for using the genetic model *MerTK^flox/flox^/Tie2^Cre^* mice with specific deletion of MerTK in ECs to study the role of EC MerTK in the development of AAAD*.*

**Figure 6:**
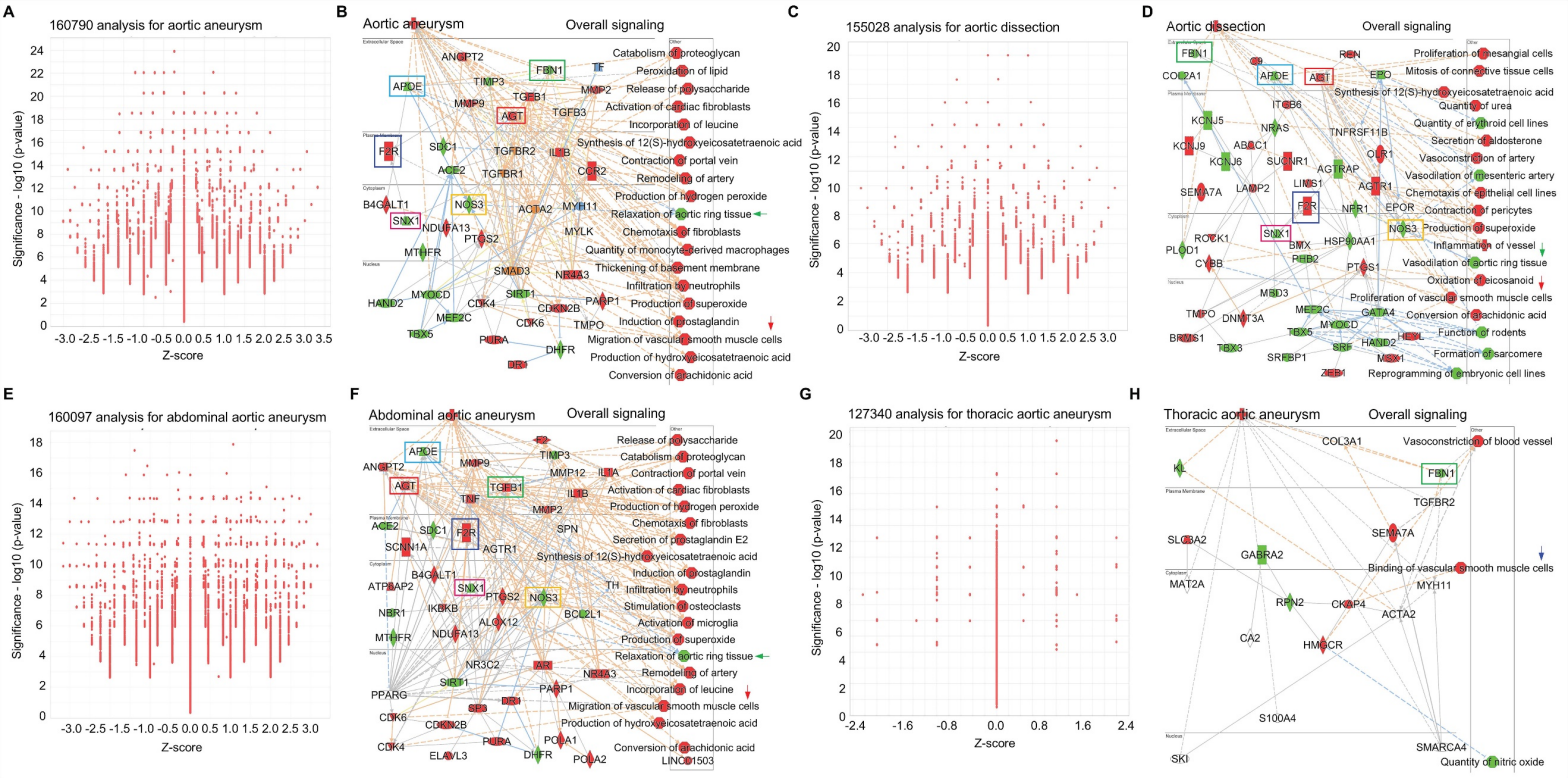
** Big data analytics for aortic aneurysm.** (**A**-**B**) Big data analytics with 160790 cross analyses for overall signaling of aortic aneurysm based on up-to-date RNA-seq data from humans, mouse and rat. In IPA of Pathways and Lists, aortic aneurysm was set as the keywords. (**C**-**D**) 155028 cross analyses for overall signaling of aortic dissection based on up-to-date RNA-seq data from humans, mouse and rat. (**E**-**F**) 160097 cross analyses for overall signaling of aortic dissection based on up-to-date RNA-seq data from humans, mouse and rat. (**G**-**H**) 127340 cross analyses for overall signaling of aortic dissection based on up-to-date RNA-seq data from humans, mouse and rat. (**B, D** and **F**) Red: increased measurement. Green: decreased measurement. Orange: predicted activation. Bule: predicted inhibition. Glos indicates activity when opposite of measurement. The lines indicate the predicated relationship (orange: leads to activation; blue: leads to inhibition; yellow: findings inconsistent with state of downstream molecule; grey: effect not predicted.

***Big data analytics for common signaling in*** **AAD*****.*** To further investigate the signaling pathways in AAD, we performed big data analytics for aortic aneurysm, aortic dissection, abdominal aortic aneurysm and thoracic aortic aneurysm using IPA RNA-seq data from both humans and rodents (mouse and rat). **Figures 6A**-**6B** are 160790-cross analyses for aortic aneurysm, showing for activated signaling (e.g., ANGPT2- Angiopoietin 2*,* F2R-coagulation factor II thrombin receptor, AGT- angiotensinogen, TGFβ1, IL1B and CCR2- C-C motif chemokine receptor 2) and inhibited signaling (e.g., APOE-apolipoprotein E, FBN1-fibrillin 1, NOS3-nitric oxide synthase 3, and SNX1-sorting nexin 1). ***Figures 6A***-**6B** also show activated mechanism pathways (e.g., peroxidation of lipid, remodeling of artery, infiltration of neutrophils and migration of SMCs) and inhibited pathway such as relaxation of aortic ring tissue. **Figures 6C**-**6D** are 155208-cross analyses for aortic dissection that show the changes of signaling (e.g., activation of AGT, F2R, ITGB6*-*integrin subunit beta 6 and OLR1-oxidized low density lipoprotein receptor 1; and inhibition of FBN1, APOE, SNX1, NOS3 and HSP90AA1-heat shock protein 90 alpha family class a member 1). Mechanism pathways analysis reveals activated pathways (e.g., proliferation of mesangial cells, vasoconstriction of artery, and proliferation of SMCs) and inhibited pathways (e.g., vasodilation of mesenteric artery, vasodilation of aortic ring tissue, and formation of sarcomere). ***Figures 6E***-**6F** are 160097-cross analyses for abdominal aortic aneurysm. We summarized the activated signaling (e.g., AGT, F2R, TGFβ1 and IL1B) and inhibited signaling (e.g., APOE, SNX1, NOS3 and BCL2L1-BCL like 1) as well as activated mechanism pathways (e.g., inflation of neutrophils, remodeling of artery, migration of SMCs and chemotaxis of fibroblasts) and inhibited mechanism pathways such as relaxation of aortic ring tissue. Finally, we performed 127340-cross analyses for thoracic aortic aneurysm as shown in **Figures 6G**-**6H**. Here we also summarized the changes of signaling (e.g., activation of SLCBA2-solute carrier family 2 member 2, SEMA7A- semaphorin 7A and CKAP4-cytoskeleton-associated protein 4; and inhibition of FBN1, KL- klotho, GABRA2*-*gamma aminobutyric acid receptor subunit alpha 2 and RPN2-ribophorin II) and the changes of mechanism pathways including activated vasoconstriction of blood vessel and binding of vascular SMCs and inhibited quantity of NO. Interestingly, we found several shared signaling pathways across these different types of AAD, such as activation of genes such as F2R and AGT and inhibition of genes such as FBN1, APOE, E, SNX1, and NOS3. In addition, we also found shared pathways related to SMC functions, such as migration, proliferation and binding, and inhibition of aortic ring tissue relaxation/vasodilation* in* AAD(**Figures 6A**-**6H**)*.*

**Figure 7:**
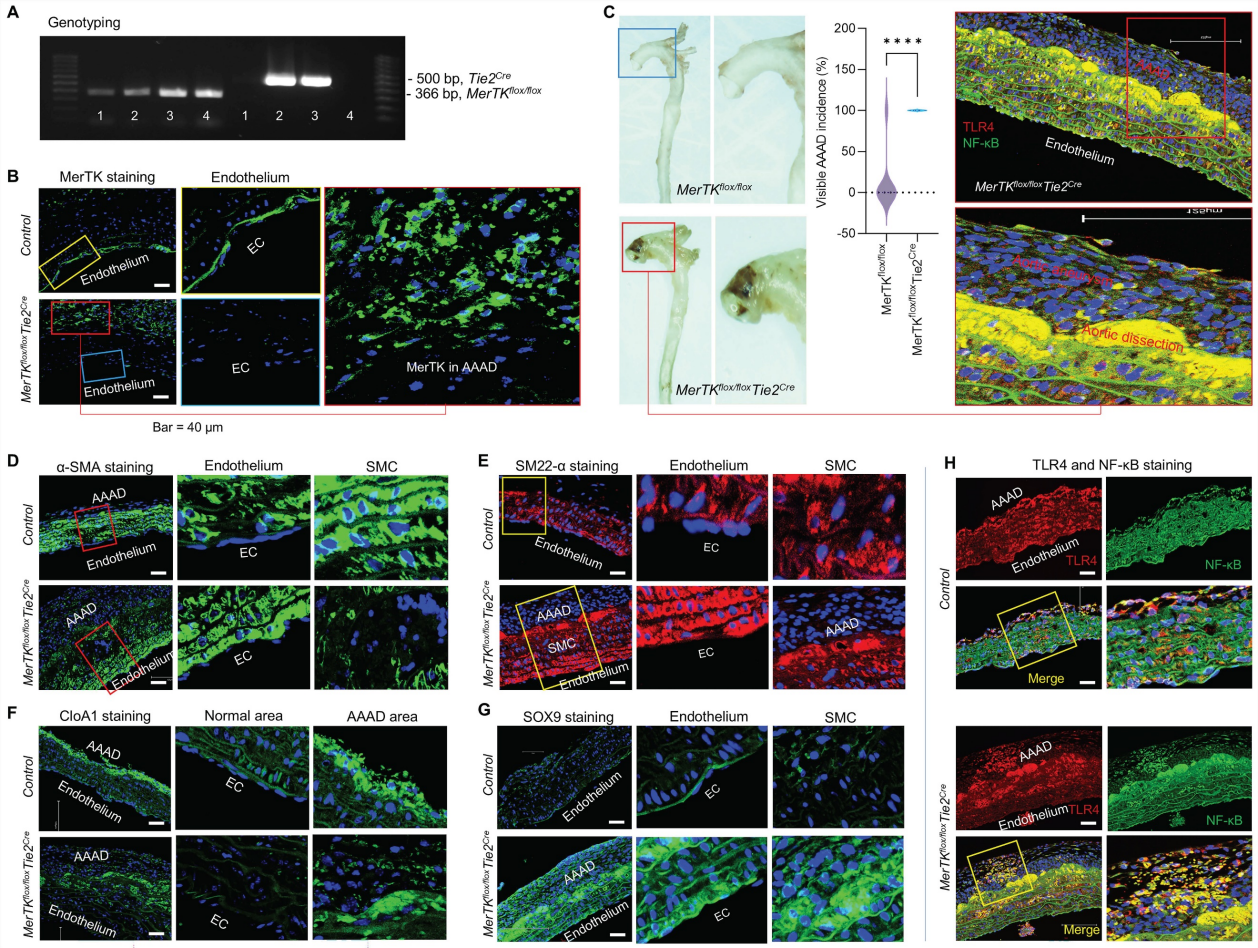
** Endothelial MerTK deficiency promotes AAAD.** (**A**) Genotyping for *MerTK^flox/flox^* and Tie2-Cre. (**B**) Immunostaining for MerTK expression in aorta from *MerTK^flox/flox^* mice and* MerTK^flox/flox^/Tie2^Cre^* mice. *(**C**)* The visible incidence rate of AAAD in ascending aorta from *MerTK^flox/flox^/Tie2^Cre^* mice (*MerTK^flox/flox^* mice crossed with Tie2-Cre mice, n=7) and littermate controls (*MerTK^flox/flox^*, n=9). An unpaired Student's t-test was used to determine statistical significance between *MerTK^flox/flox^* mice and* MerTK^flox/flox^*/Tie2^Cre^ mice. Data was analyzed with GraphPad Prism 9.4.1 and summarized as the mean ± SD. ****P<0.0001. (**D**-**H**) Immunostaining for expression of α-SMA, SM22α, CloA1, SOX9, TLR4 and NF-κB in ascending aorta from AAAD model of *MerTK^flox/flox^*/Tie2-Cre mice (n=7) and *MerTK^flox/flox^* littermate controls (n=9).

**MerTK deficiency in ECs promotes AAAD.** To validate the big data analytics and scRNA-seq data in humans, we generated a unique genetic model (*MerTK^flox/flox^/Tie2^Cre^ mice)* with MerTK deletion in ECs by crossing* MerTK^flox/flox^* mice with Tie2-Cre mice, as shown by our genotyping results (**Figure 7A**).* MerTK^flox/flox^/Tie2^Cre^* mice and their littermate controls (*MerTK^flox/flox^*) were infused with saline or Ang II (1000 ng·kg^-1^·min^-1^) for 7 days to set up the AAAD model. As shown in **Figure 7B**, MerTK is highly expressed in endothelium compared with other areas in the ascending aorta of *MerTK^flox/flox^* control mice. As expected, there is no MerTK expression in endothelium from *MerTK^flox/flox^/Tie2^Cre^* mice. Interestingly, a significant increase of MerTK expression was found in AAAD areas compared to other areas of *MerTK^flox/flox^/Tie2^Cre^* mice. This suggests the high non-EC efferocytosis activities in AAAD, mainly from macrophages or other monocytes, an indicator for increasing accumulation of apoptotic cells and subsequent vascular deterioration. Our data showed that the visible incidence rate of AAAD in *MerTK^flox/flox^/Tie2^Cre^* mice is significantly higher than that of littermate controls (100% vs. 11.1%, **Figure 7C**). α-SMA and SM22α are the most used markers of contractile SMCs for the detection of endothelial to mesenchymal transition* (*EndMT, an indicator for endothelial dysfunction). Our data showed that, compared with littermate controls, *MerTK^flox/flox^/Tie2^Cre^* mice displayed significantly larger AAAD areas that are visible almost in the whole ascending aorta* (***Figure 7D**-**E***).* In contrast, there is only a small AAAD area in the littermate controls. More importantly, α-SMA* and* SM22α are mainly expressed in SMCs with almost no detection in the endothelium of littermate controls* (***Figure 7D**-**E***).* However, the expression of α-SMA* and* SM22α is markedly increased in the endothelium while sharply decreased in SMCs of AAAD area in the *MerTK^flox/flox^/Tie2^Cre^* mice. This indicates that endothelial MerTK deficiency promotes EndMT and SMC phenotypic alterations. The SMC phenotypic alterations are also associated with increased secretion of collagen type I. Our immunostaining data showed that, compared to normal areas without AAAD, collagen type I alpha 1 (CloA1) is highly expressed in AAAD areas in both *MerTK^flox/flox^/Tie2^Cre^ mice* and their littermate controls (**Figure 7F**). Sex-determining region Y box 9 (SOX9) is another marker for SMC phenotypic alterations.^28^ Interestingly, we found that SOX9 is primarily expressed at higher levels in the endothelium of the littermate controls. In *MerTK^flox/flox^/Tie2^Cre^* mice, SOX9 expression is markedly increased in endothelium, SMCs and AAAD areas (**Figure 7G**). The toll-like receptor 4 (TLR4)/NF-κB axis is a known inflammatory pathway that promotes endothelial inflammation and SMC pro-inflammatory phenotype. Our data indicates that TLR4/NF-κB is extensively expressed throughout the aorta in both *MerTK^flox/flox^/Tie2^Cre^* mice and littermate controls (**Figure 7H**). However, TLR4/NF-κB expression is much higher in AAAD areas compared to normal areas without AAAD.

## Discussion

AAD occurs with the formation of a tear within the aortic wall, causing blood to flow between the laminar layers of the media, and subsequently separating them and creating a false lumen with a severely weakened outer aortic wall 1,8. This condition presents a high risk of the aortic rupture, as the aortic walls expand, leading to a mortality of ~80% 1,8. Unfortunately, no effect clinical treatments have been developed to treat AAD 1. Increasing evidence showed that endothelial dysfunction, characterized by impaired EC efferocytosis, increased oxidative stress and enhanced endothelial inflammation, plays a central role in AAAD development 1,14-16. Our recent studies have shown that MerTK deficiency induces impaired efferocytosis in aortic ECs, increased expression of NADPH oxidases (a main source of cellular ROS- reactive oxygen species) and elevated levels of endothelial inflammation markers (e.g., cleaved IL-1B, TLR4 and NF-κB) 14-16. Based on this, we posited that endothelial MerTK may play a key role in AAAD development. This study was designed to clarify whether endothelial MerTK plays a role in the pathogenesis of AAAD and to provide a foundation for further mechanism studies. Our main findings include the central role of aortic ECs in AAAD, revealed by scRNA-seq analysis in human AAAD patients, the potential involvement of endothelial MerTK in AAAD identified by multi-comparative scRNA-seq analyses, the shared signaling pathways across different types of AAAD uncovered by big data analytics, and the detrimental impact of endothelial MerTK on AAAD development demonstrated using a genetic model with endothelial MerTK deficiency. Our data provides insights into the roles of ECs and endothelial MerTK in the pathogenesis of AAAD.

Accumulation of apoptotic cells and the ensuing inflammatory and immune responses are the common features of AAAD 29,30. Apoptotic cells are usually cleared by professional phagocytes such as macrophages through a process called efferocytosis 11. However, recent studies have shown that aortic ECs have a high ability to perform efferocytosis via MerTK in atherosclerosis and vascular aging, two diseases closely associated with AAAD 12-16. Inhibition of endothelial MerTK induces the accumulation of apoptotic cells and exacerbates the pro-inflammatory response, highlighting the potential role of endothelial MerTK in AAAD 1,12-16. To investigate the specific role of ECs in AAAD and explore the potential mechanisms of endothelial MerTK-mediated AAAD, we performed a comparative analysis of ECs with other cells (e.g., SMCs, macrophages and NK cells) using scRNA-seq analysis from human AAAD. Our data showed that, compared to SMCs, ECs are the main source of pro-inflammatory cytokines, including IL-1A, IL-1B, TNF, IL-6, and IFNL1. In contrast to macrophages, ECs have a lower capacity for phagocytosis, a cellular process for clearing pathogens and apoptotic cells also known as efferocytosis. Additionally, when compared to NK cells, ECs promote SMC migration, enhance cell adhesion, and drive endothelial tube formation, along with the secretion of a series of pro-inflammatory cytokines (e.g., TGFB1, IL-6 and IL1B). In summary, our comparative analysis of scRNA-seq in human AAD reveals that ECs secret a variety of pro-inflammatory cytokines, which contribute to SMC phenotypic alterations and the activation of macrophages and NK cells. Impaired EC phagocytosis, including efferocytosis, may be important for AAAD development. Notably, we discovered that EC engulfment of other cells, such as presenting cells, phagocytes, leukocytes, blood cells and myeloid cells, is inhibited in AAAD. This inhibition is associated with a decreased MerTK level, suggesting dysfunctional EC efferocytosis as a potential mechanism in AAAD.

However, there are several limitations in scRNA-seq and comparative analyses in human AAAD. First, AAAD can occur at different stages, such as acute or chronic; but based on provided patient information for ascending aortic samples 8, we were unable to differentiate between these stages. Second, variations in medical conditions and therapies may have influenced the data analysis. Hypertension was the only common medical condition within these patients and controls, while other conditions such as chronic obstructive pulmonary disease, aortic valve regurgitation, bicuspid aortic valve and reoperation differed between them 8. Third, wall shear stress, an important hemodynamic reaction involved in AAAD development via vascular wall remodeling 32, could not be assessed in AAAD tissues, a common challenge in studies examining hemodynamics and AAAD formation. Fourth, the small sample size in human studies limits the statistical power, potentially explaining the lack of significance in some upstream regulators and pathways identified in comparative analysis, including canonical pathways, disease and biofunctions, and tox functions. Finally, the AAAD and control tissues were not collected simultaneously, which may have affected the tissue processing times and introduced variability. Further studies should aim to recruit a larger cohort of AAAD patients with similar stages, medical conditions and therapies, and consistent tissue processing times.

To validate the findings from scRNA-seq in human AAAD and big data analytics that indicate the key role of endothelial MerTK in AAAD, we used *MerTK^flox/flox^* mice and *MerTK^flox/flox^Tie2^Cre^* mice to establish an AAAD model through a 7-day Ang II infusion. We provided the first evidence to support the role of endothelial MerTK in development of AAAD, since we observed a significant difference in the incidence of AAAD between *MerTK^flox/flox^/Tie2^Cre^* mice and their littermate controls (100% vs. 11.1%). Further studies are warranted to characterize the functional changes of macrophages, SMCs, NK cells, mast cells, monocytes, MSCs, and plasma B cells, as well as their cell-cell interactions during the development of AAAD. There are limitations in our mouse studies. One limitation of this study is that the 7-day Ang II infusion represents an early AAAD model. Investigating more advanced AAAD models, such as those with a 28-day or longer Ang II infusion, is necessary to further understand the disease progression. Another limitation is that the underlying mechanisms of endothelial MerTK in AAAD require further exploration. Through the big data analytics, we identified the shared signaling across different types of AAD, including activated pathways (e.g., AGT, IL1B, F2R, and TGFB1) and inhibited pathways (e.g., APOE, FBN1, NOS3, and SNX1). These shared signaling pathways may represent novel mechanisms in endothelial MerTK-mediated AAAD that need to be further validated.

In summary, using scRNA-seq analysis and big data analytics in humans, along with our unique AADD model established in *MerTK^flox/flox^/Tie2^Cre^* mice, we have discovered endothelial MerTK deficiency as a novel mechanism underlying AAAD development. We have also identified specific cellular pathways focused on endothelial MerTK, which may open new avenues for potential therapeutic interventions designed to modulate endothelial MerTK in AAAD. In addition, our study may also have clinical relevance for other endothelial MerTK-associated cardiovascular diseases, such as atherosclerosis and vascular aging.

## Supplementary Material

Supplementary information.
