# Supplementary figures and images for: Big data analytics and scRNA-seq in human aortic aneurysms and dissections: role of endothelial MerTK
Source: Theranostics. 2025 Jan 1;15(1):202–15. doi: 10.7150/thno.103851 (PMC11667232; doi:10.7150/thno.103851)

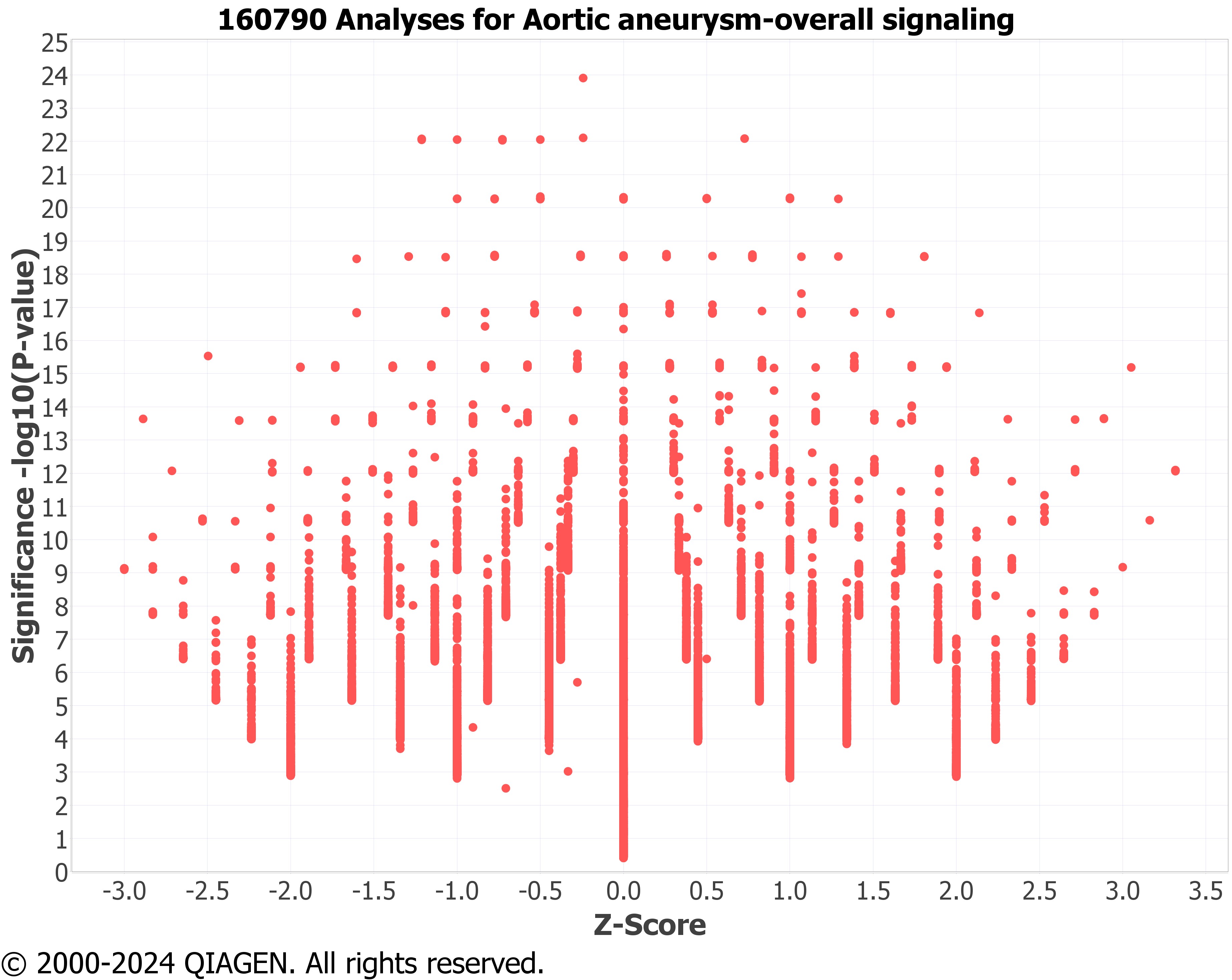

Supplement: Supplementary file 1 — Supplementary information. [file thnov15p0202s1.zip › 6-Large data analytics for AAD/AAD overal signaling--160790 anallysis-1.jpg]

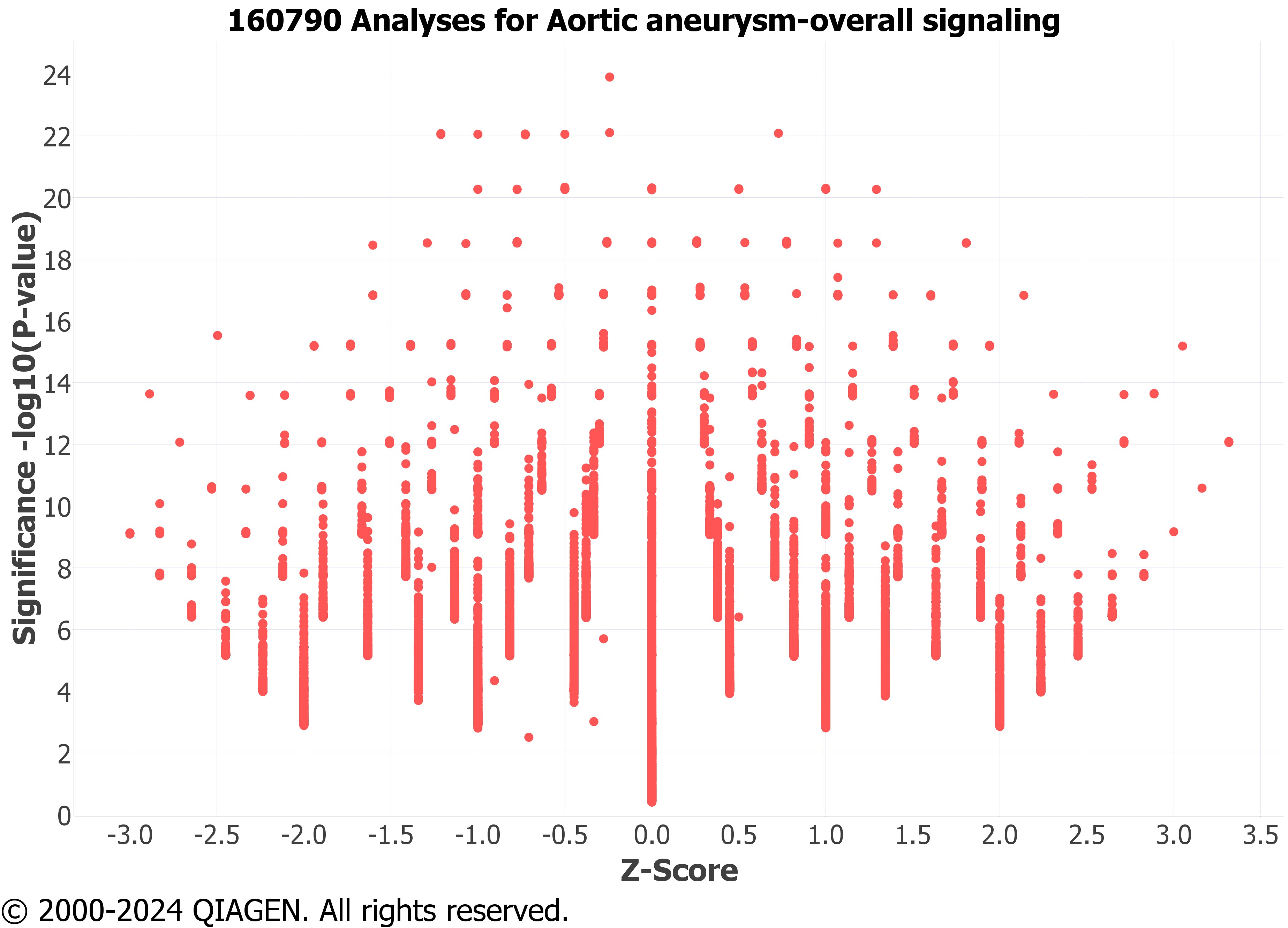

Supplement: Supplementary file 1 — Supplementary information. [file thnov15p0202s1.zip › 6-Large data analytics for AAD/AAD overal signaling--160790 anallysis.jpg]

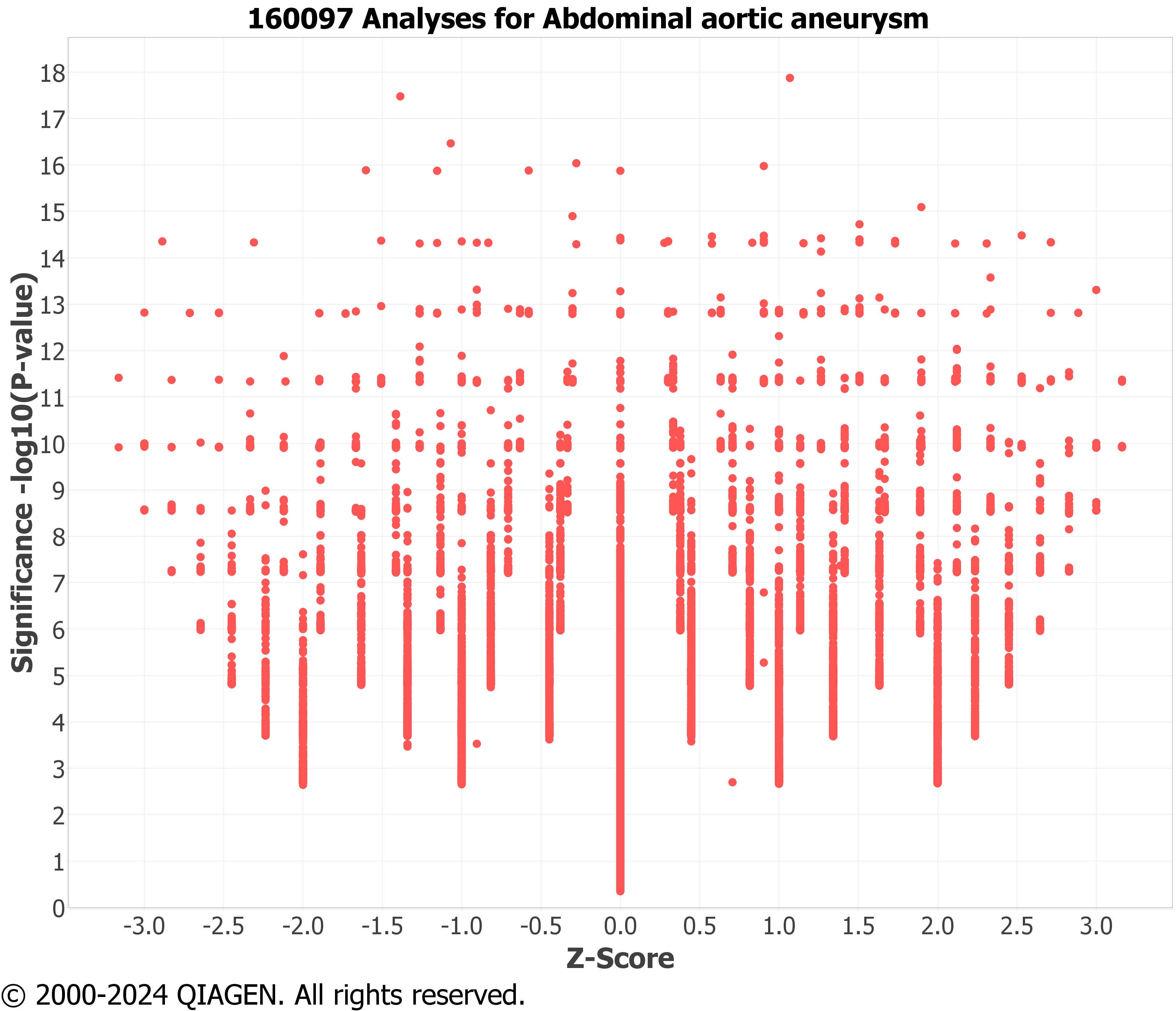

Supplement: Supplementary file 1 — Supplementary information. [file thnov15p0202s1.zip › 6-Large data analytics for AAD/Abdominal aortic aneurysm -1600097 anallysis.jpg]

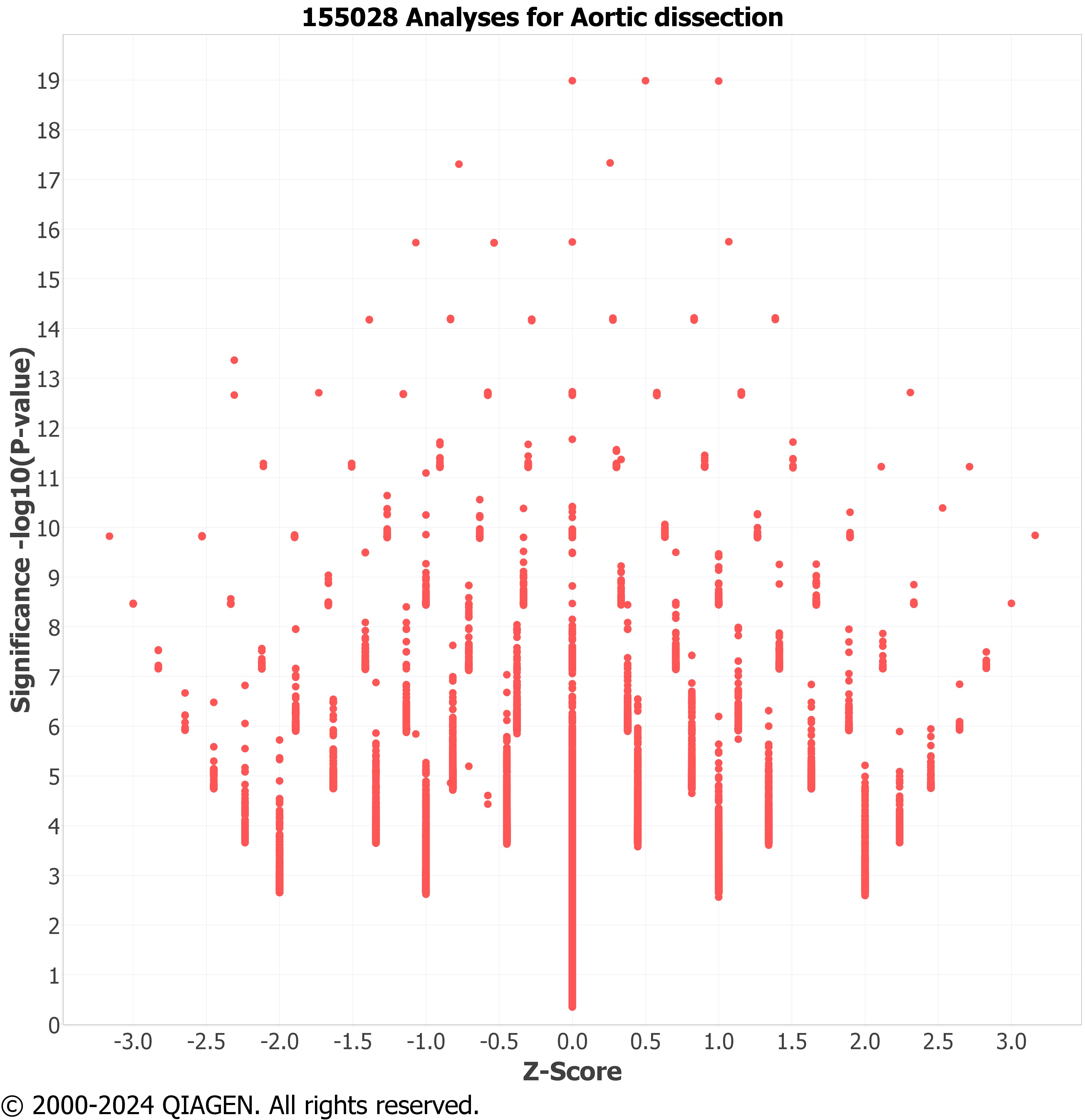

Supplement: Supplementary file 1 — Supplementary information. [file thnov15p0202s1.zip › 6-Large data analytics for AAD/Aortic dissection -150028 anallysis.jpg]

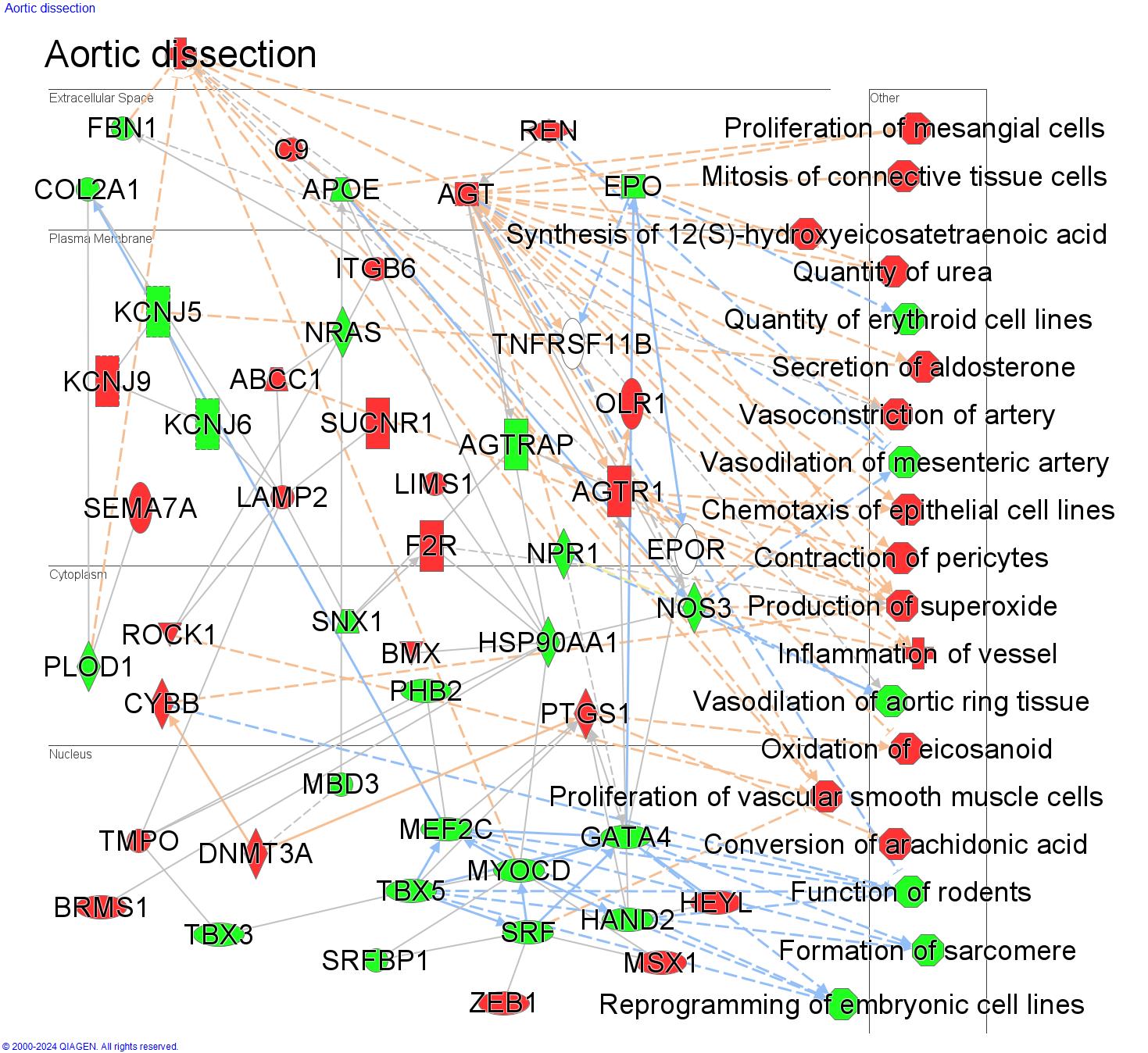

Supplement: Supplementary file 1 — Supplementary information. [file thnov15p0202s1.zip › 6-Large data analytics for AAD/Aortic dissection signaling.jpg]

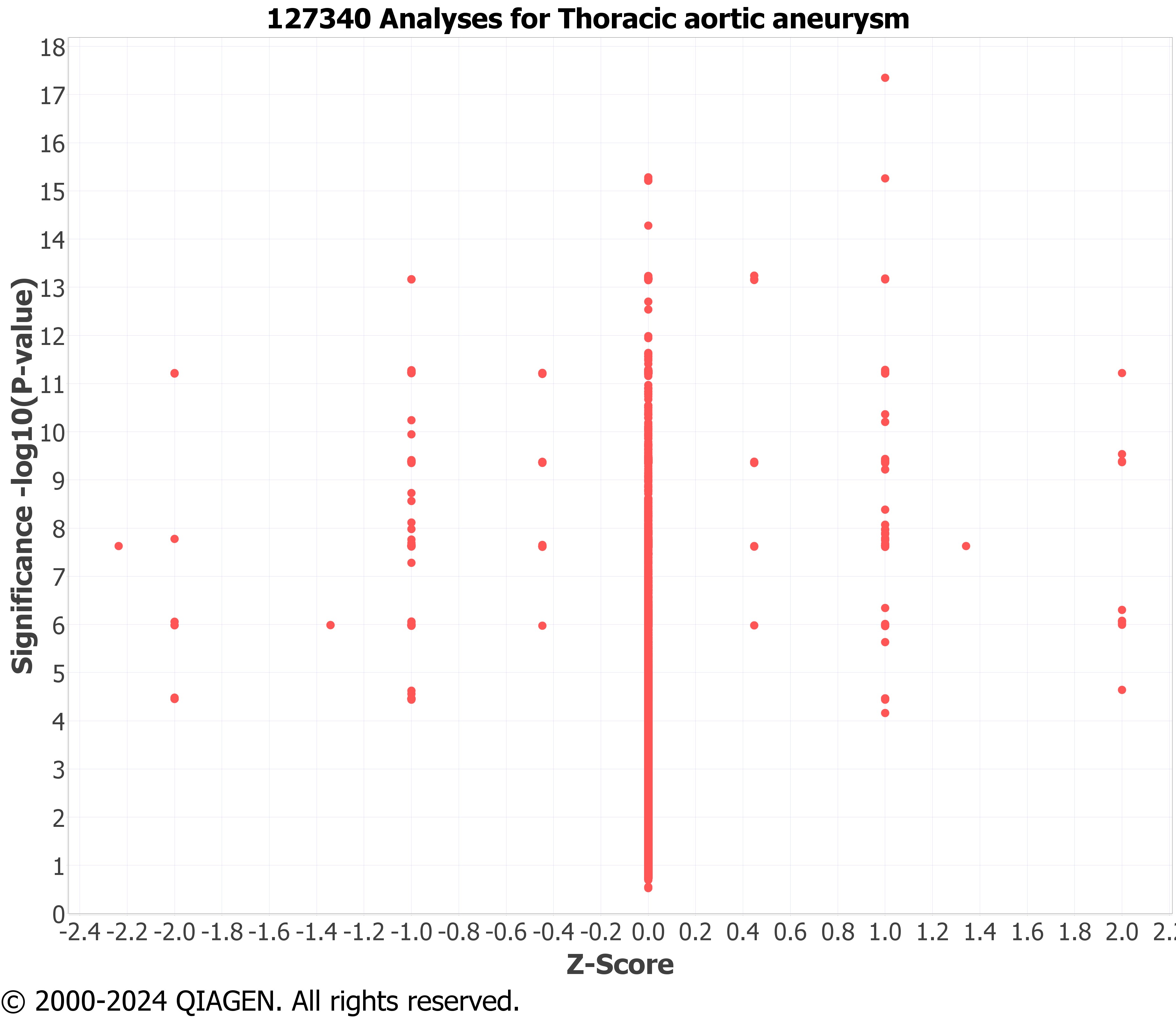

Supplement: Supplementary file 1 — Supplementary information. [file thnov15p0202s1.zip › 6-Large data analytics for AAD/Thoracic aortic aneurysm-127340 anallysis.jpg]

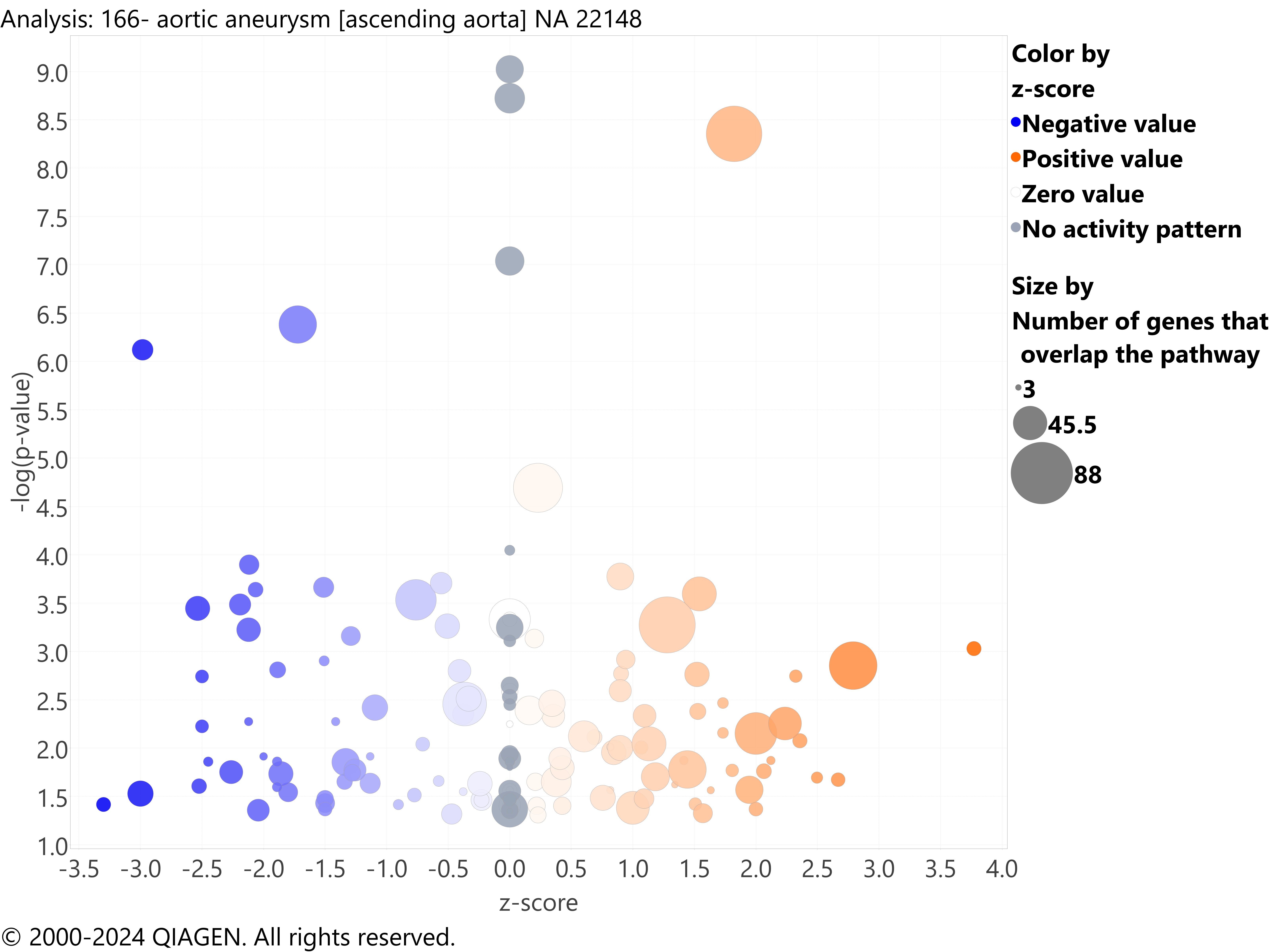

Supplement: Supplementary file 1 — Supplementary information. [file thnov15p0202s1.zip › 4-EC cluster vs other cells/Canonical pathways-EC clusters vs other cells.jpg]

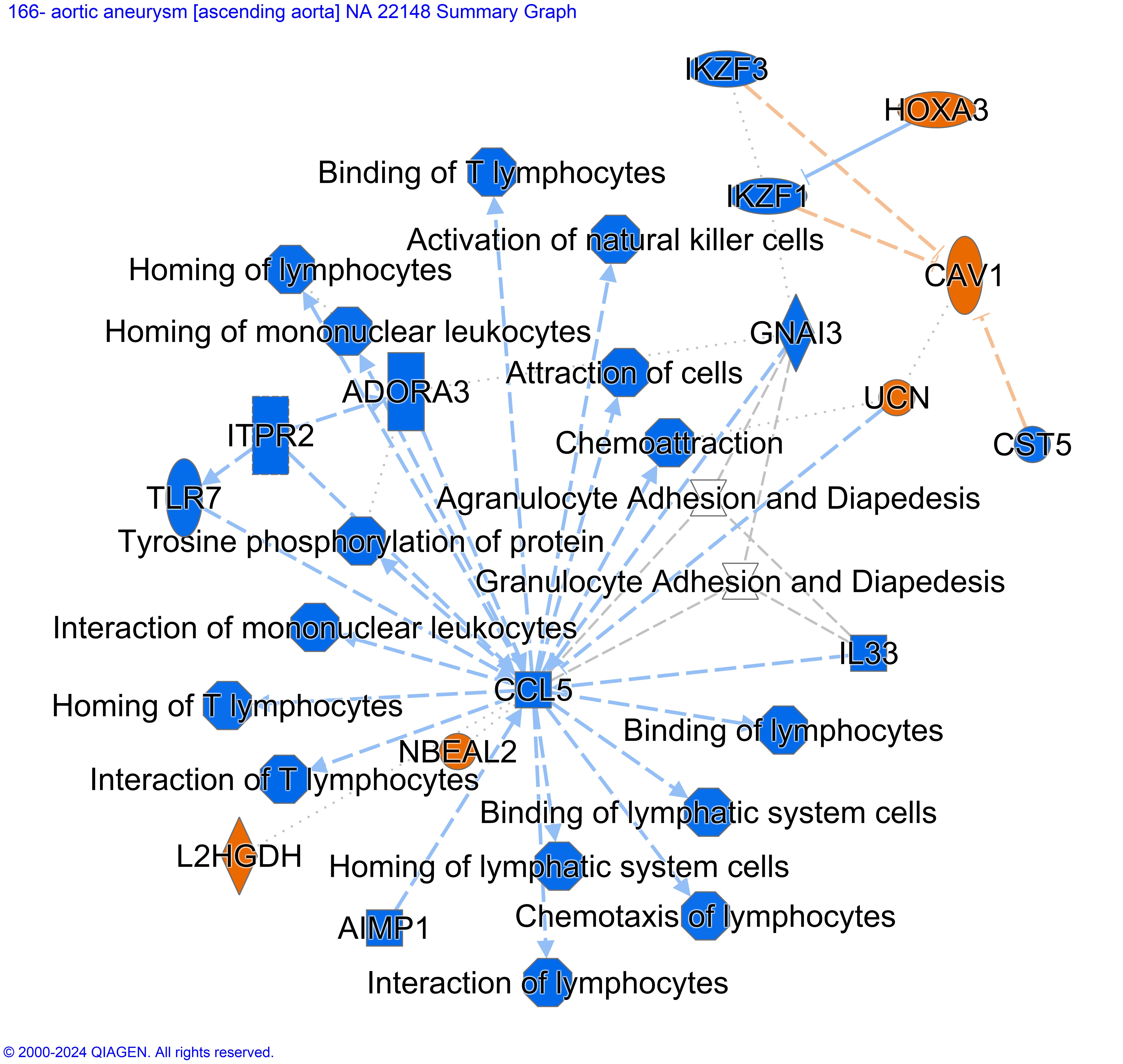

Supplement: Supplementary file 1 — Supplementary information. [file thnov15p0202s1.zip › 4-EC cluster vs other cells/Graphical summary-EC clusters vs other cells.jpg]

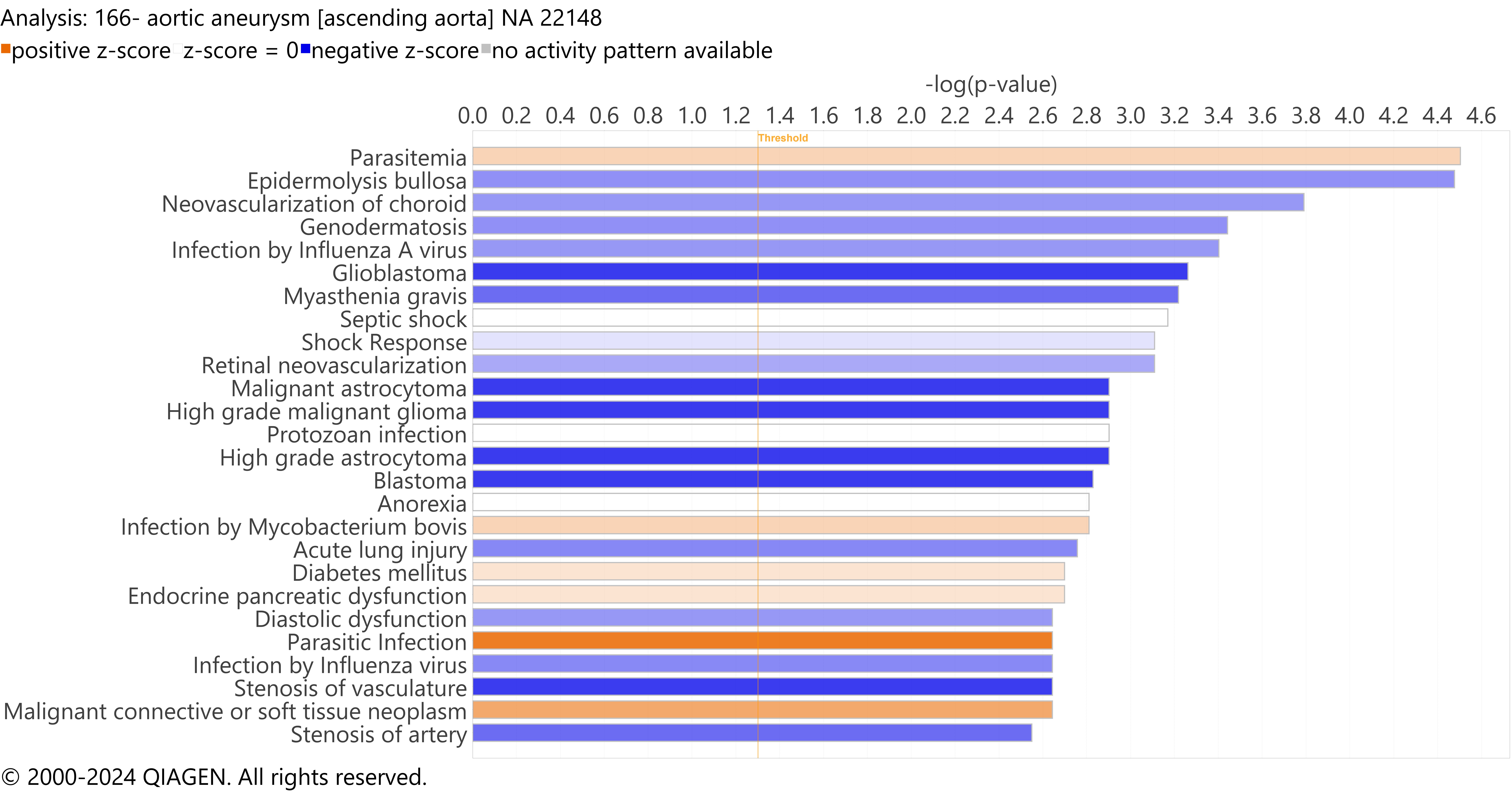

Supplement: Supplementary file 1 — Supplementary information. [file thnov15p0202s1.zip › 4-EC cluster vs other cells/ML disease pathways-EC clusters vs other cells.jpg]

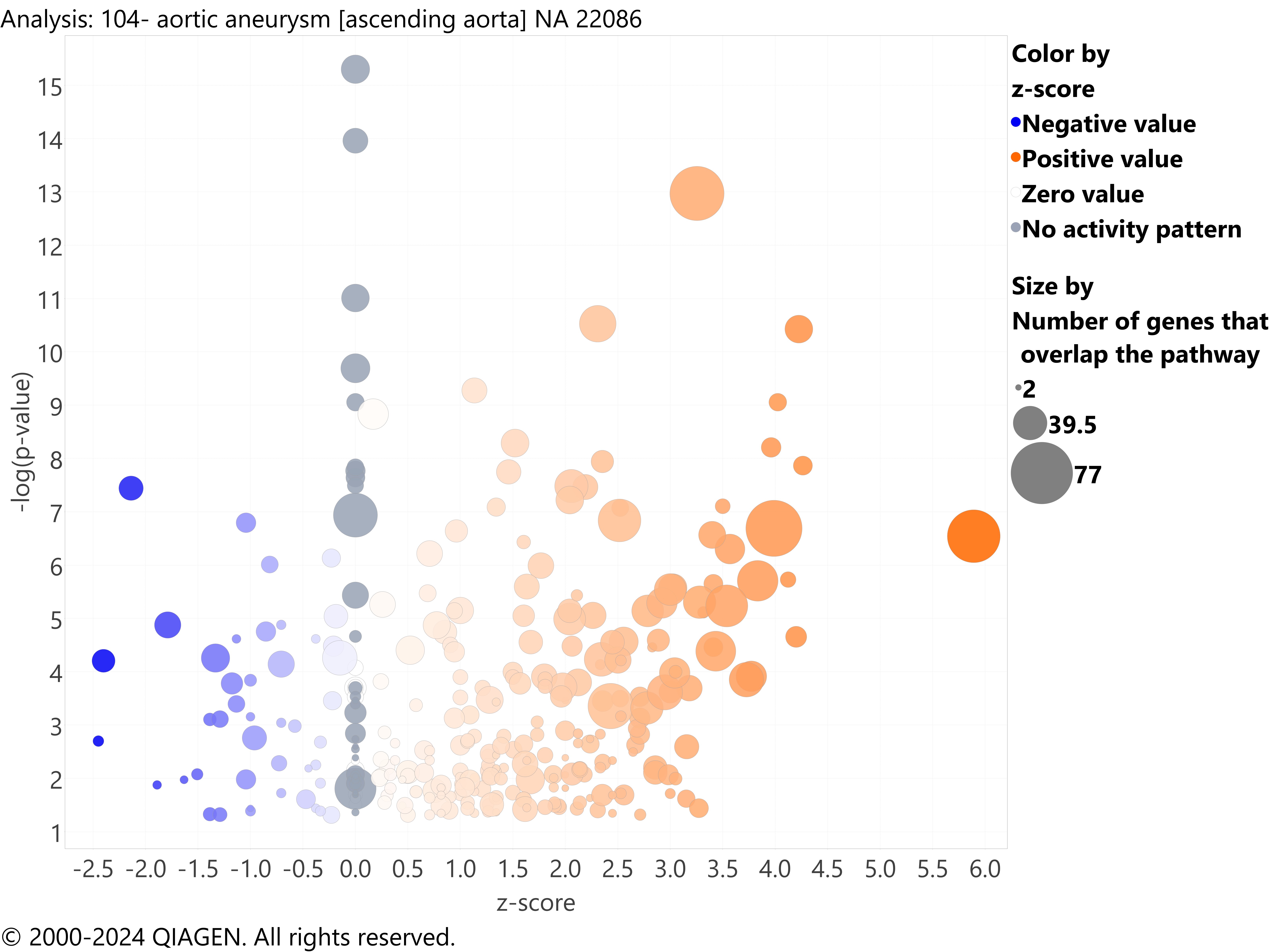

Supplement: Supplementary file 1 — Supplementary information. [file thnov15p0202s1.zip › 3-EC vs NK cell/Canonical Pathways-EC vs NK cell.jpg]

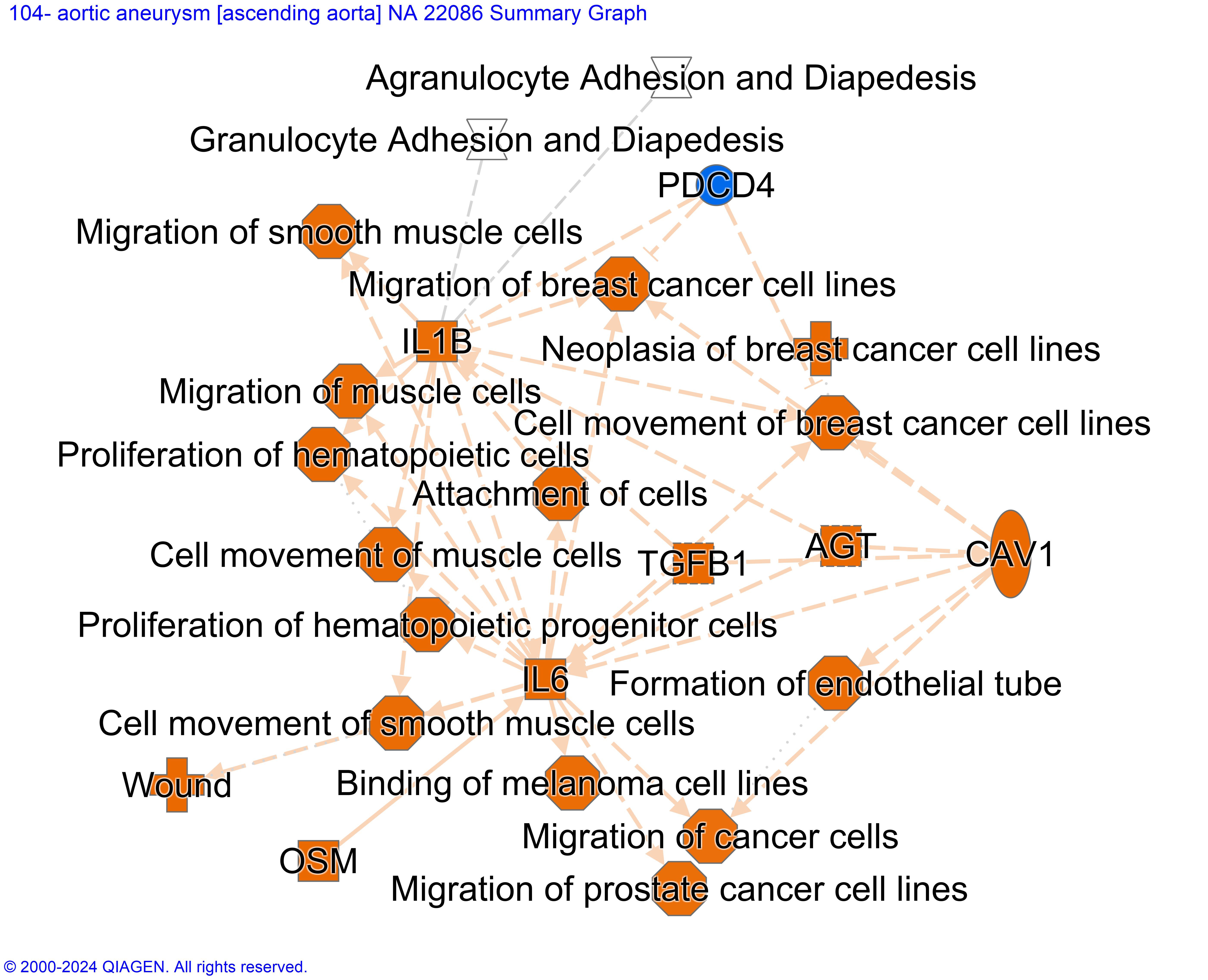

Supplement: Supplementary file 1 — Supplementary information. [file thnov15p0202s1.zip › 3-EC vs NK cell/Graphical Summary-EC vs NK cell.jpg]

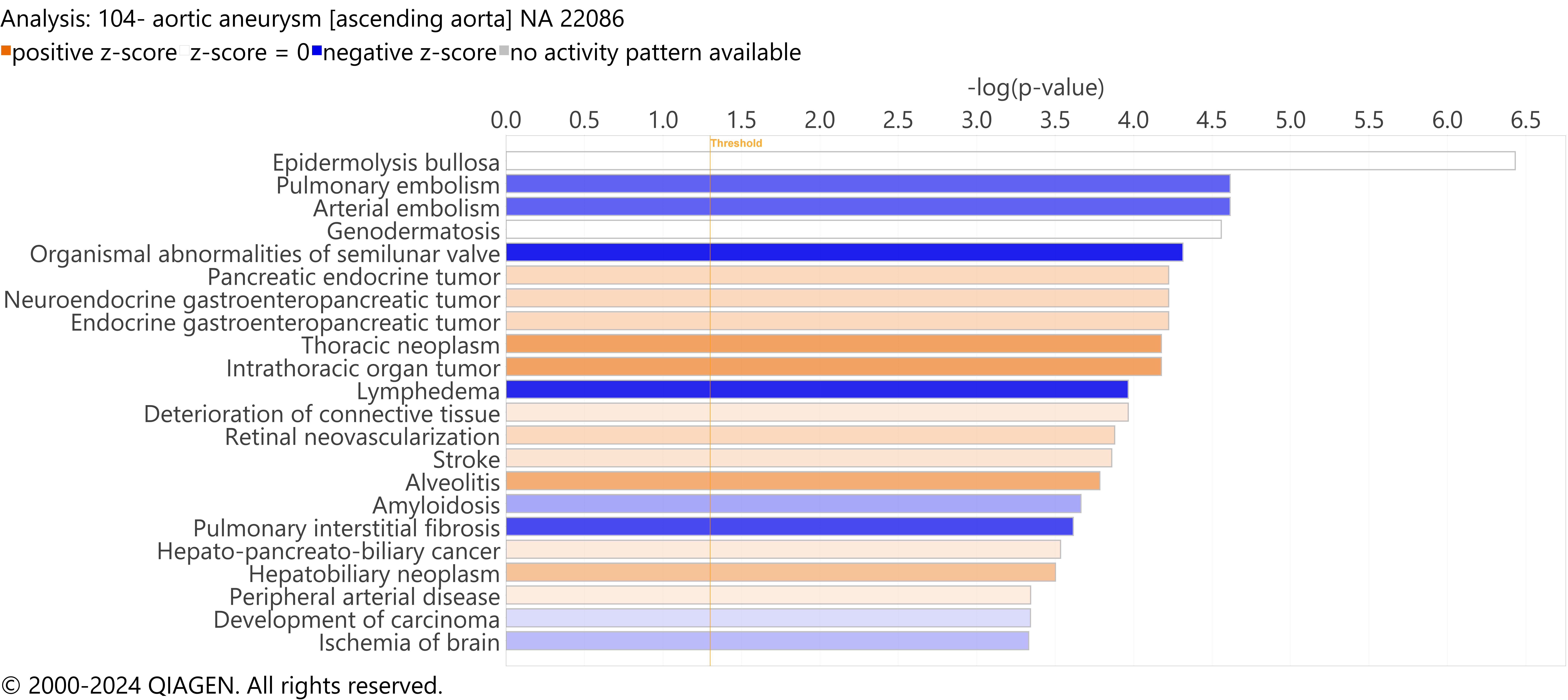

Supplement: Supplementary file 1 — Supplementary information. [file thnov15p0202s1.zip › 3-EC vs NK cell/ML Disease Pathways-EC vs NK cell.jpg]

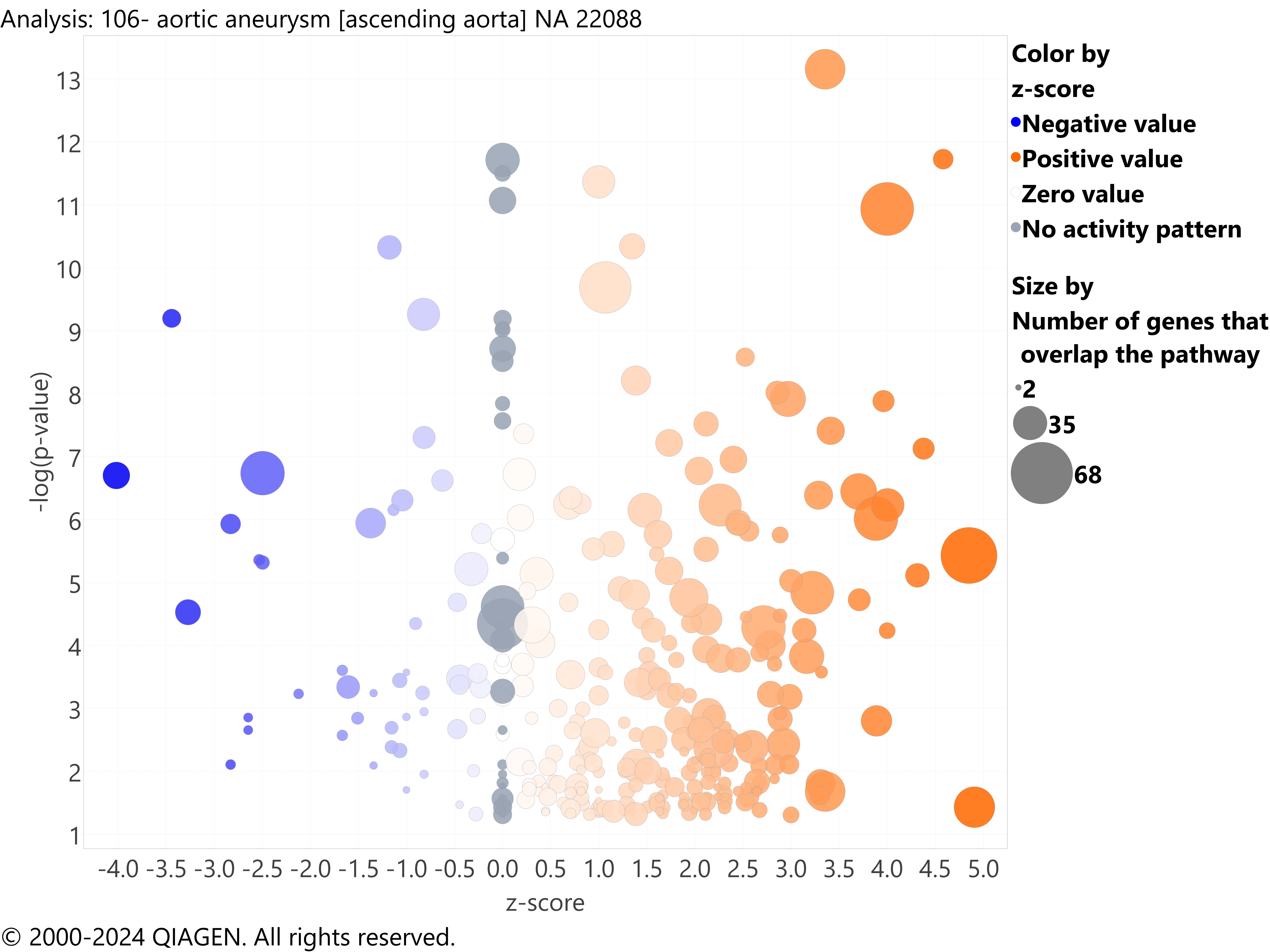

Supplement: Supplementary file 1 — Supplementary information. [file thnov15p0202s1.zip › 2-EC vs SMC/Canonical Pathways-EC vs SMC.jpg]

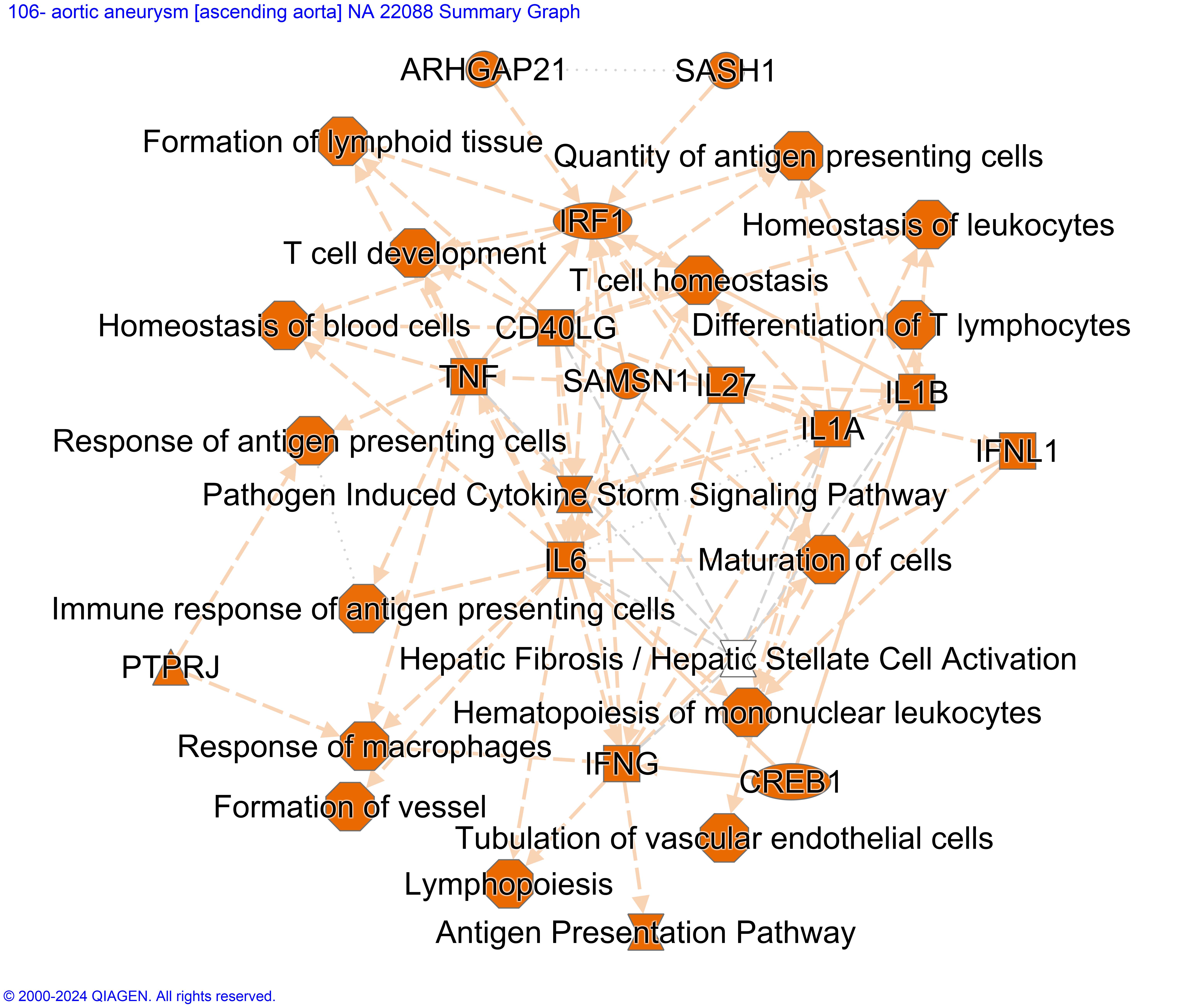

Supplement: Supplementary file 1 — Supplementary information. [file thnov15p0202s1.zip › 2-EC vs SMC/Graphical Summary.jpg]

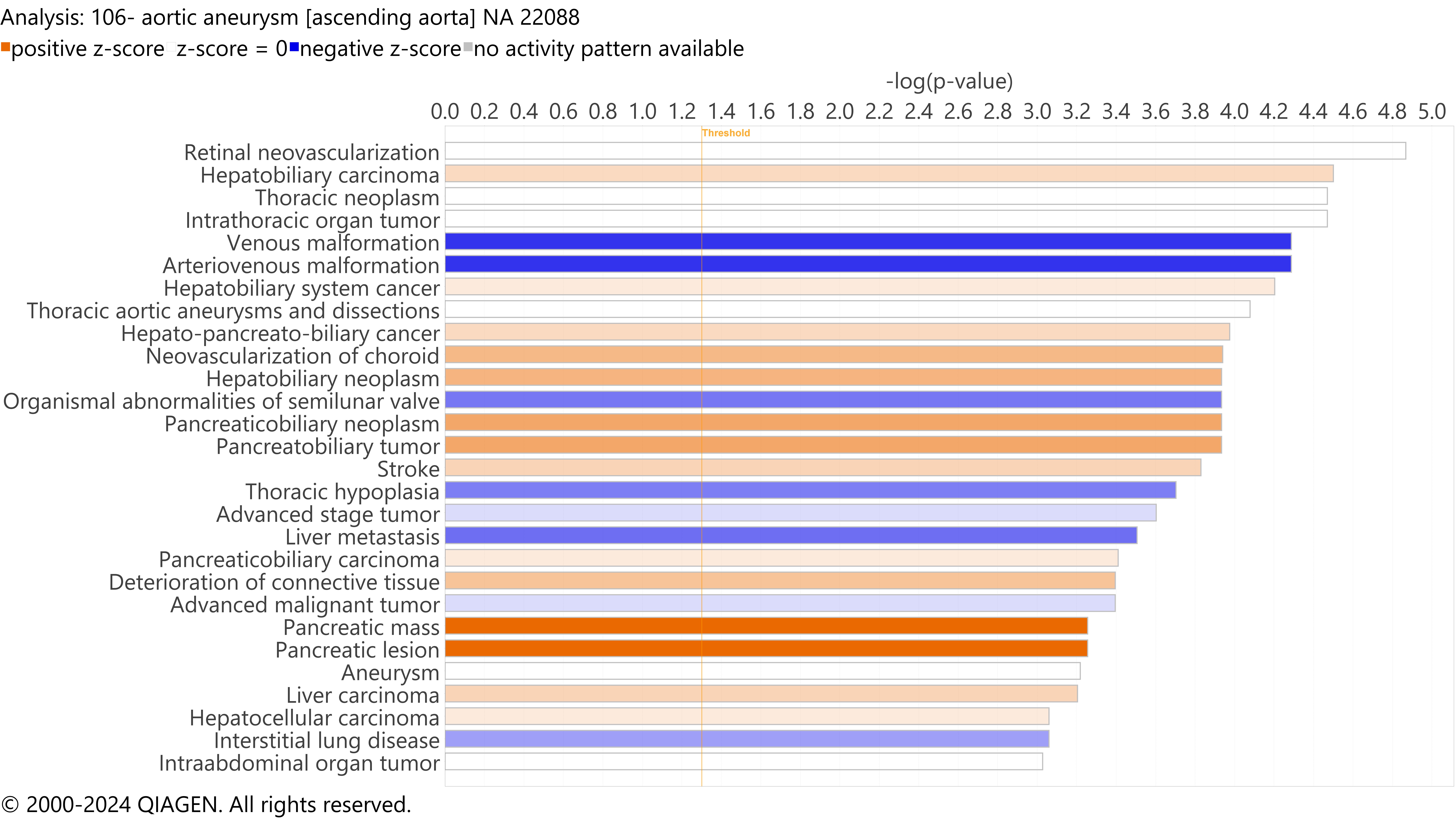

Supplement: Supplementary file 1 — Supplementary information. [file thnov15p0202s1.zip › 2-EC vs SMC/ML Disease Pathways-1-EC vs SMC.jpg]

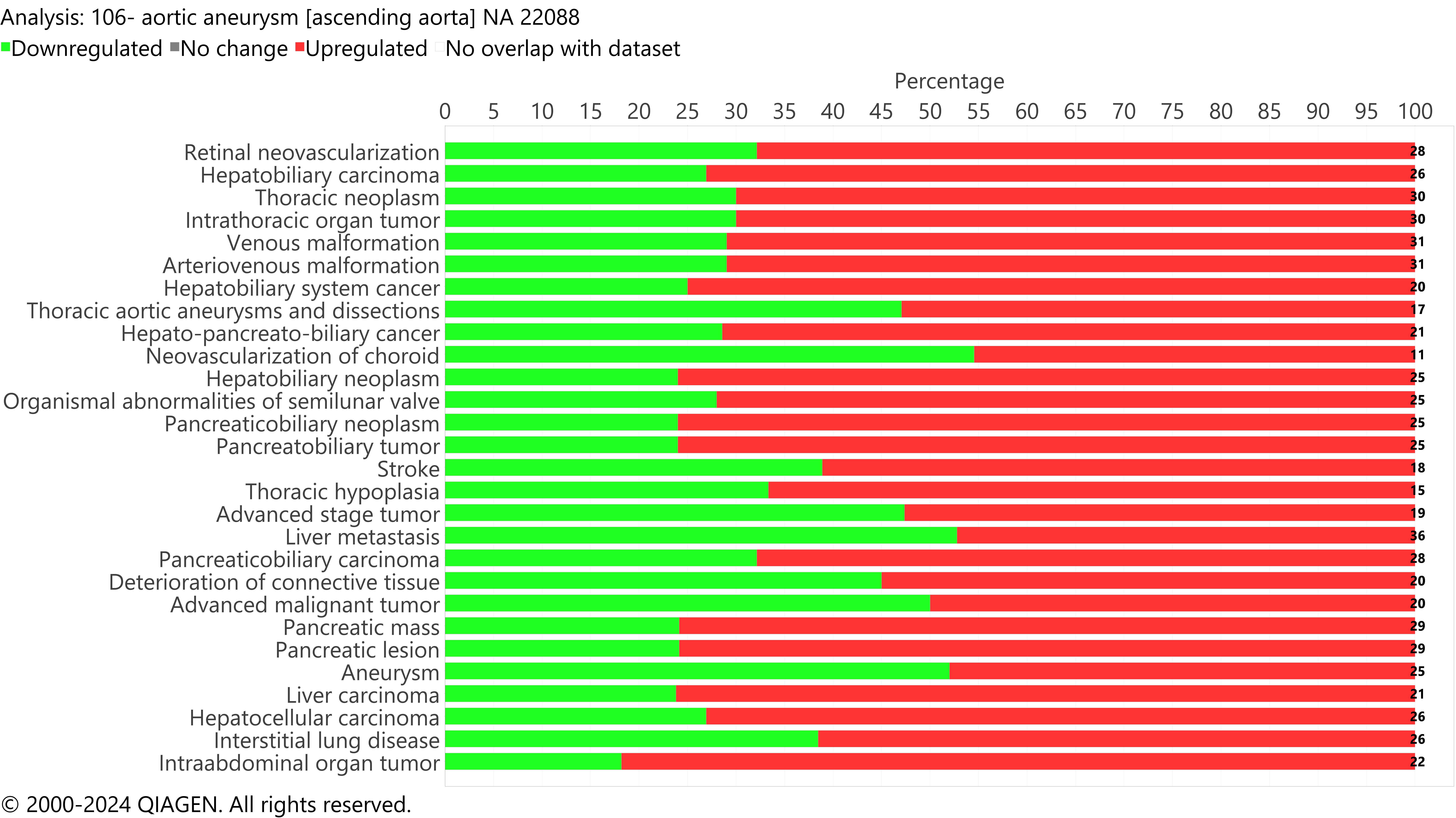

Supplement: Supplementary file 1 — Supplementary information. [file thnov15p0202s1.zip › 2-EC vs SMC/ML Disease Pathways-EC vs SMC.jpg]

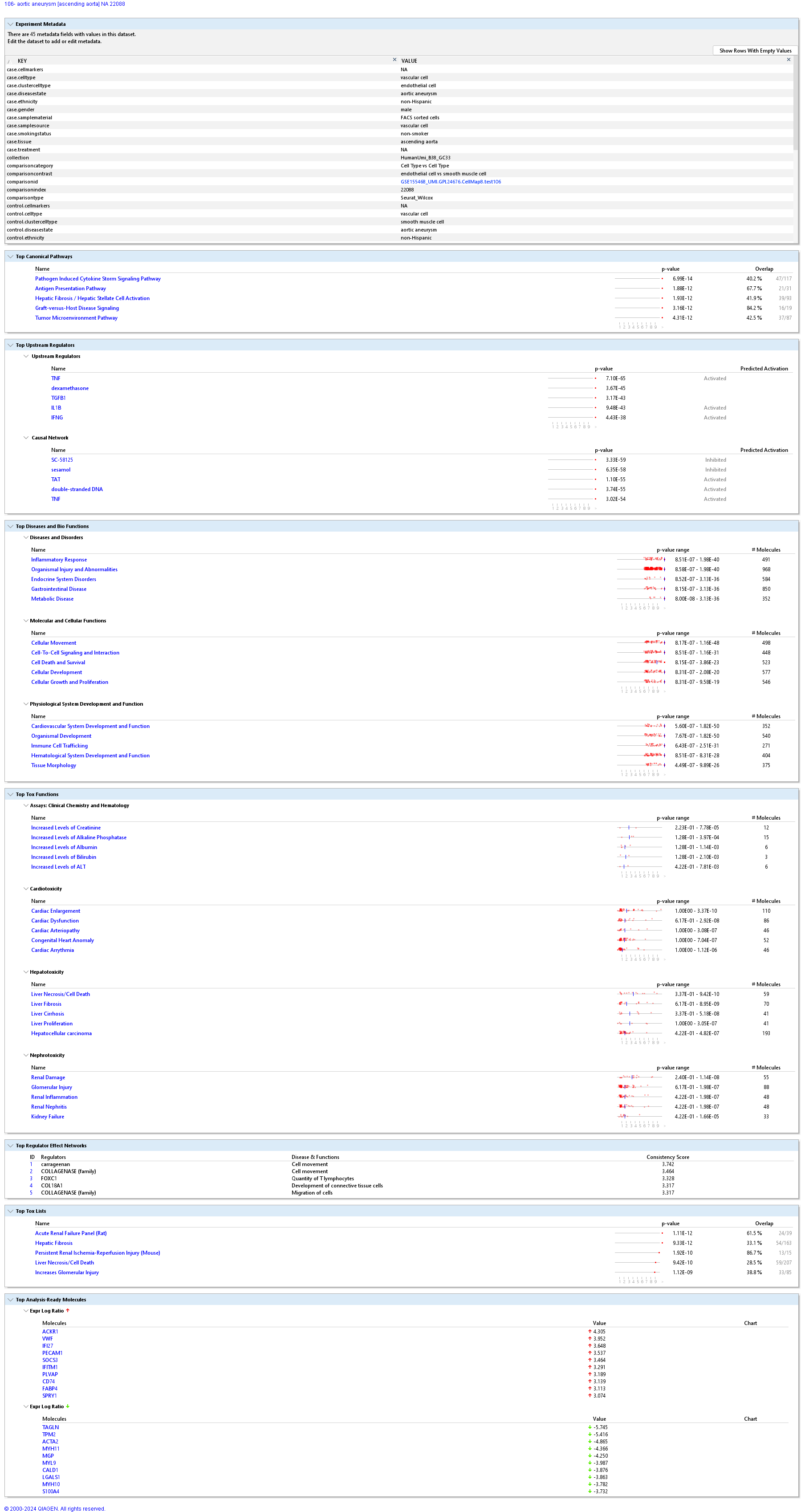

Supplement: Supplementary file 1 — Supplementary information. [file thnov15p0202s1.zip › 2.1 EC vs SMC/Summary.png]

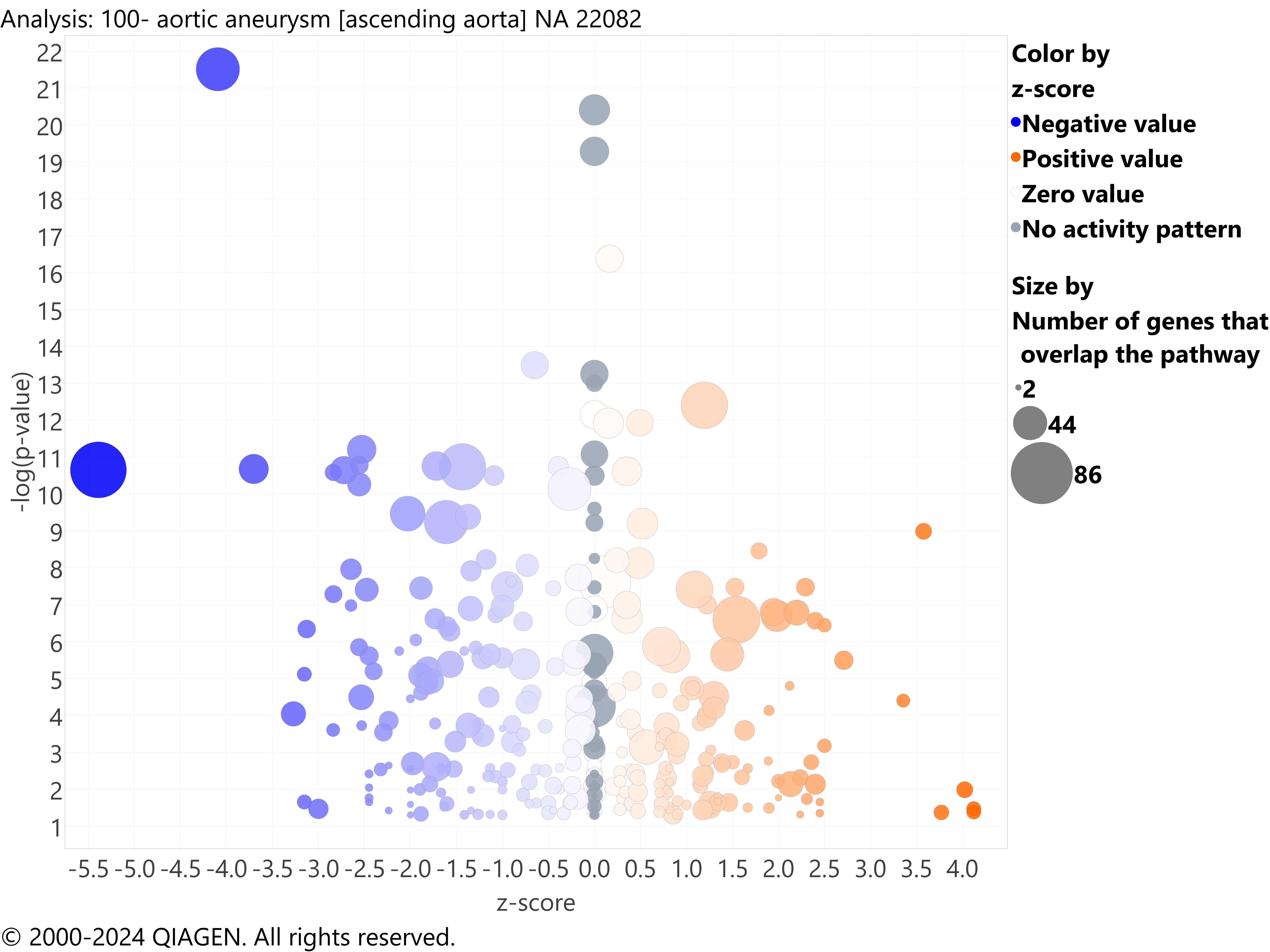

Supplement: Supplementary file 1 — Supplementary information. [file thnov15p0202s1.zip › 1-EC vs macrophage/Canonical Pathways-EC vs Macrophage.jpg]

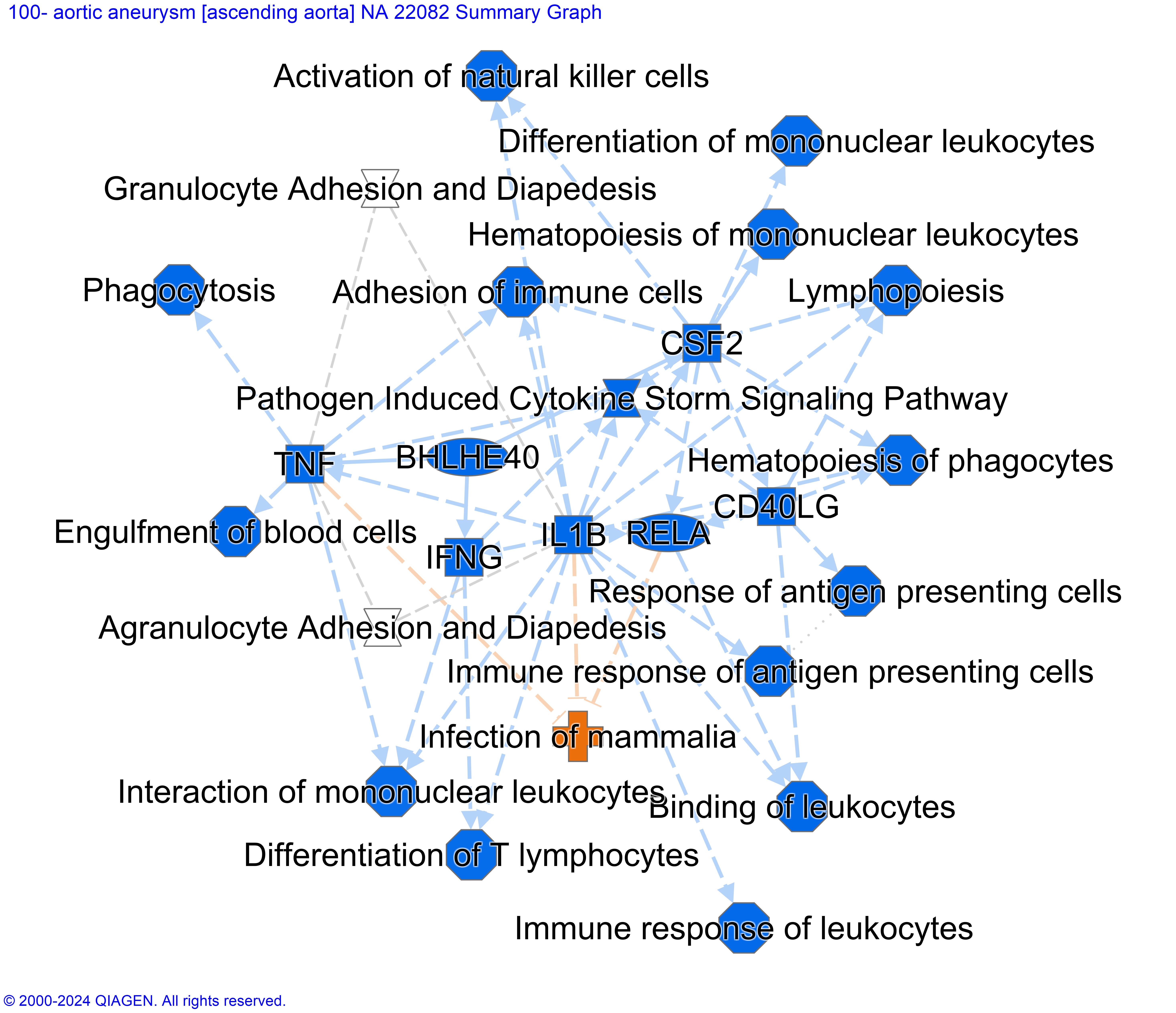

Supplement: Supplementary file 1 — Supplementary information. [file thnov15p0202s1.zip › 1-EC vs macrophage/Graphical Summary-EC vs Macrophage.jpg]

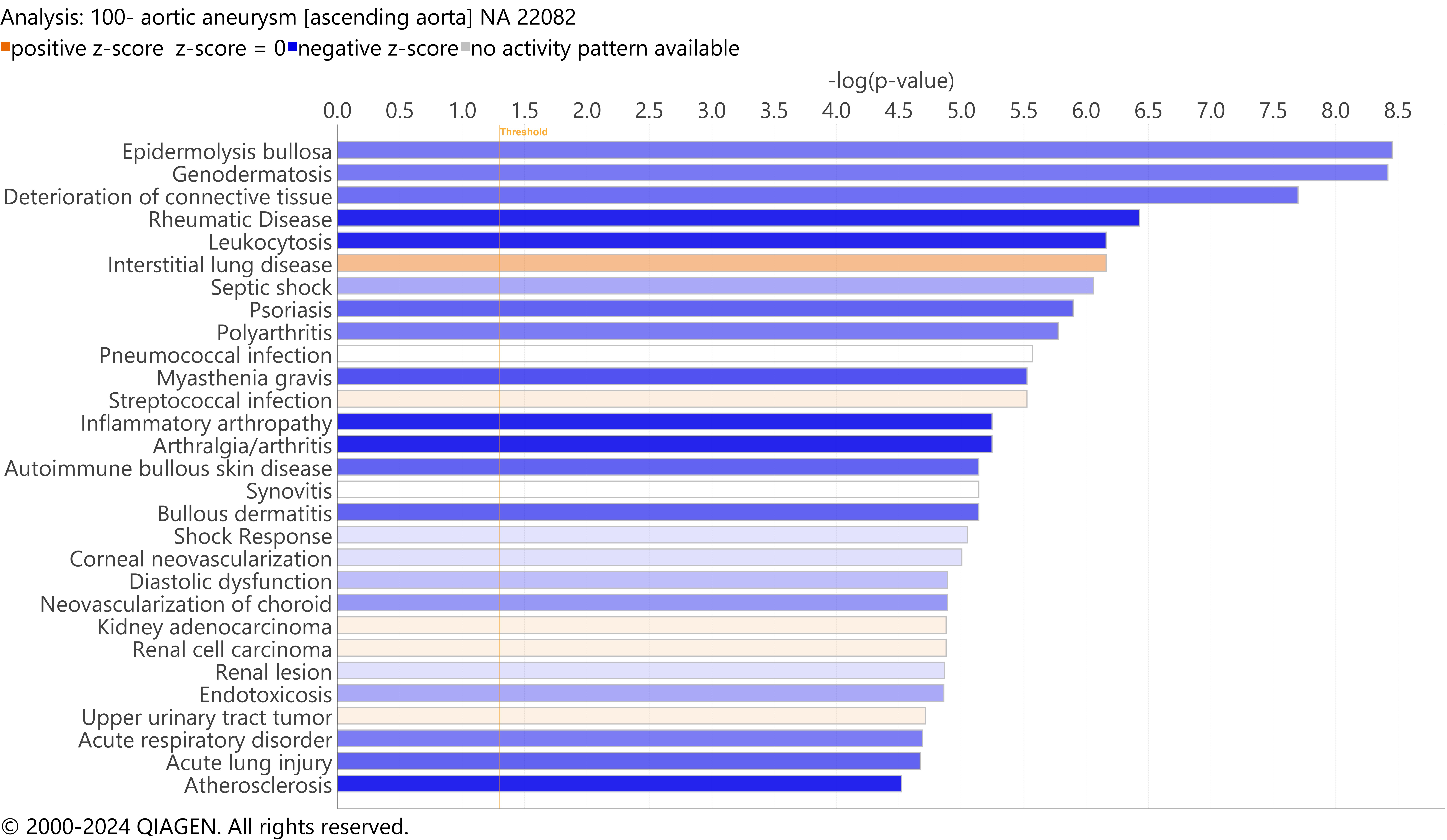

Supplement: Supplementary file 1 — Supplementary information. [file thnov15p0202s1.zip › 1-EC vs macrophage/ML Disease Pathway-EC vs Macrophage.jpg]
